# Supplementary material for: Overcoming Chemical, Biological, and Computational Challenges in the Development of Inhibitors Targeting Protein-Protein Interactions
Source: Chem Biol. 2015 Jun 18;22(6):689–703. doi: 10.1016/j.chembiol.2015.04.019 (PMC4518475; doi:10.1016/j.chembiol.2015.04.019)
Supplement: Document S1. Figure S1 and Table S1 [file mmc1.pdf]

Chemistry & Biology, Volume *22*

Supplemental Information

Overcoming Chemical, Biological, and Computational Challenges in the Development of Inhibitors Targeting Protein-Protein Interactions

Luca Laraia, Grahame McKenzie, David R. Spring, Ashok R. Venkitaraman, and David J. Huggins

Supporting Information

Supplemental Data

Figure S1, Related to Figure 1

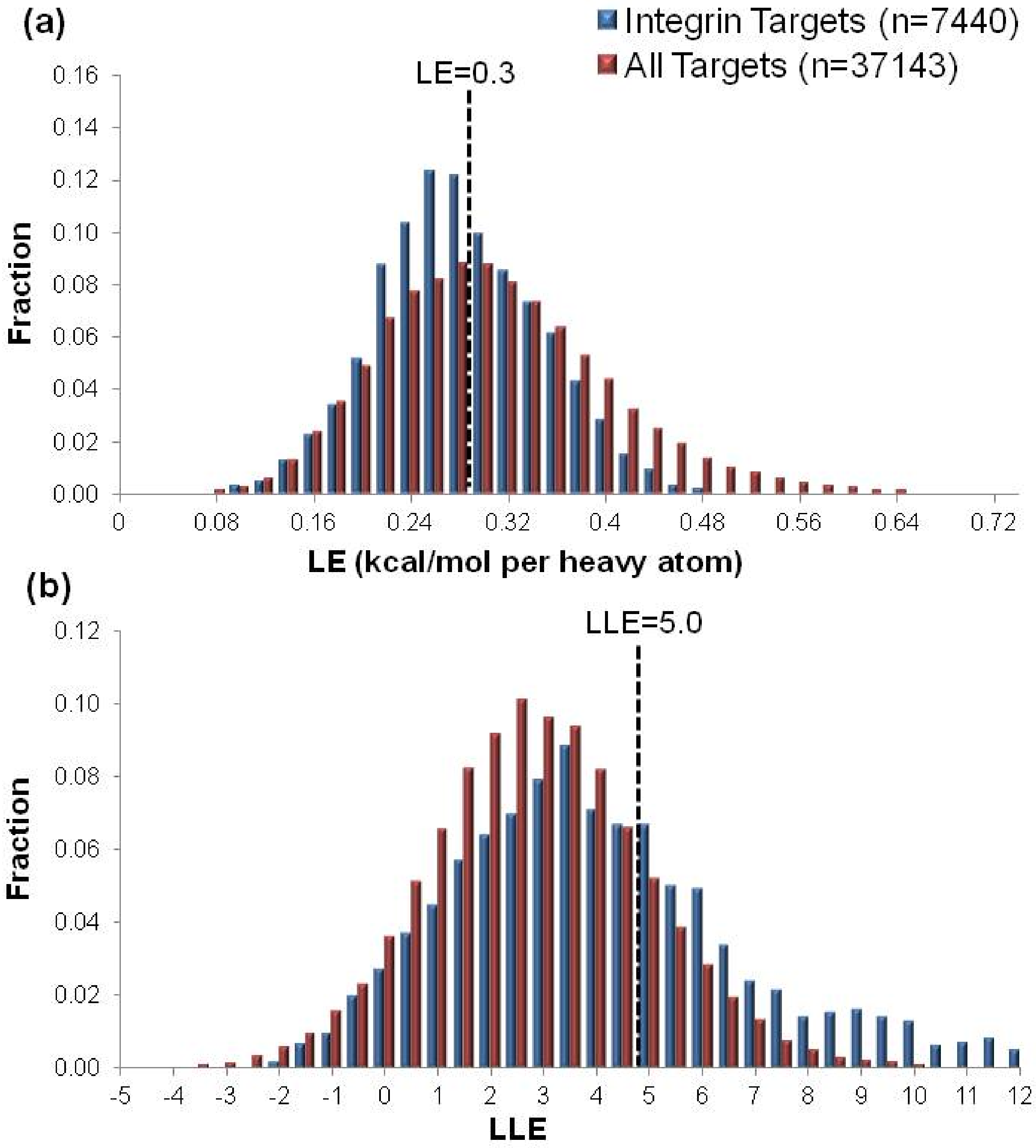

Figure S1 - Bar graphs showing the distributions of (a) LE and (b) LLE using IC_{50} data for 7440 integrin inhibitors in the TIMBAL database and 37143 small molecule inhibitors in the curated portion of the BindingDB database. Heavy atom counts and cLogP values were computed using Schrödinger's Qikprop and the small molecules were prepared using Schrödinger's Ligprep. The average LE for the integrin inhibitors is 0.29 kcal/mol per heavy atom, compared with an average of 0.23 kcal/mol per heavy atom for the other PPI inhibitors in TIMBAL and 0.32 kcal/mol per heavy atom for inhibitors in the BindingDB. The average LLE for the PPI inhibitors studied was 4.38, compared with an average of 1.32 for the other PPI inhibitors in TIMBAL and 3.12 for inhibitors in the BindingDB. The percentages of integrin inhibitors in the TIMBAL database, other PPI inhibitors in the TIMBAL database and inhibitors in the BindingDB passing the LE filter are 42.9%, 14.5%, and 54.8% respectively. The percentages of integrin inhibitors in the TIMBAL database, other PPI inhibitors in the TIMBAL database and inhibitors in the BindingDB passing the LLE filter are 35.8%, 4.5%, and 17.4% respectively.

Table S1

Technique	Acronym	Description	Software
Molecular Mechanics - Generalized Born Surface Area (Massova and Kollman, 2000)	MM-GBSA	A method for computing the free energy difference between two states by computing the free energies using forcefield based energies and a Generalized Born implicit solvent model.	NAMD (Phillips, et al., 2005)<br>AMBER (Case, et al., 2005)<br>GROMACS (Van Der Spoel, et al., 2005)<br>CHARMM (Brooks, et al., 2009)<br>GROMOS (Christen, et al., 2005)<br>Desmond (Bowers, et al., 2006)
Free Energy Perturbation (Liu, et al., 2012)	FEP	A statistical mechanical method for computing the free energy difference between two states by calculating the sum of free energy changes for a series of small steps along the pathway between them.	NAMD,<br>AMBER,<br>GROMACS,<br>CHARMM,<br>GROMOS,<br>Desmond
Thermodynamic Integration (Lawrenz, et al., 2012)	TI	A statistical mechanical method for computing the free energy difference between two states by integrating over the enthalpy changes along the pathway between them.	NAMD,<br>AMBER,<br>GROMACS,<br>CHARMM,<br>GROMOS,<br>Desmond
Inhomogeneous Fluid Solvation Theory (Lazaridis, 2000)	IFST	A statistical mechanical method for computing the free energy difference between two states by calculating the effect of the change on the surrounding solvent.	STOW (Li and Lazaridis, 2012)<br>WaterMap (Young, et al., 2007)<br>GIST (Nguyen, et al., 2012)

Virtual Screening (Scior, et al., 2012)	VS	A method for identifying potential inhibitors of a given protein from computational analysis of a large library of molecules. The most common approaches are shape-based screening, pharmacophore screening and molecular docking,	Glide (Hippertt, et al., 2001)<br>GOLD (Verdonk, et al., 2003)<br>DOCK (Moustakas, et al., 2006)<br>ROCS (Rush, et al., 2005)
Replica Exchange Molecular Dynamics (Rao and Caflisch, 2003)	REMD	A technique that enhances MD sampling by performing parallel simulations of a system at multiple temperatures and allowing the different systems to exchange.	NAMD,<br>AMBER,<br>GROMACS,<br>CHARMM,<br>GROMOS,<br>Desmond

Table S1 - A description of some of the computational techniques discussed in this paper.

Supplementary References

Bowers, K.J., Chow, E., Xu, H., Dror, R.O., Eastwood, M.P., Gregersen, B.A., Klepeis, J.L., Kolossvary, I., Moraes, M.A., and Sacerdoti, F.D. (2006). Scalable algorithms for molecular dynamics simulations on commodity clusters. In *SC 2006 Conference, Proceedings of the ACM/IEEE*. (IEEE). pp. 43-43.

Brooks, B.R., Brooks, C.L., 3rd, Mackerell, A.D., Jr., Nilsson, L., Petrella, R.J., Roux, B., Won, Y., Archontis, G., Bartels, C., Boresch, S., et al. (2009). CHARMM: the biomolecular simulation program. J. Comput. Chem. 30, 1545-1614.

Case, D.A., Cheatham, T.E., Darden, T., Gohlke, H., Luo, R., Merz, K.M., Onufriev, A., Simmerling, C., Wang, B., and Woods, R.J. (2005). The Amber biomolecular simulation programs. J. Comput. Chem. 26, 1668-1688.

Christen, M., Hünenberger, P.H., Bakowies, D., Baron, R., Bürgi, R., Geerke, D.P., Heinz, T.N., Kastenholz, M.A., Kräutler, V., and Oostenbrink, C. (2005). The GROMOS software for biomolecular simulation: GROMOS05. J. Comput. Chem. 26, 1719-1751.

Hippertt, J., Rocha, A., Lana, C., Egydio-Silva, M., and Takeshita, T. (2001). Quartz plastic segregation and ribbon development in high-grade striped gneisses. J Struct Geol 23, 67-80.

Lawrenz, M., Wereszczynski, J., Ortiz-Sánchez, J.M., Nichols, S.E., and McCammon, J.A. (2012). Thermodynamic integration to predict host-guest binding affinities. J. Comput.-Aided Mol. Des. 26, 569-576.

Lazaridis, T. (2000). Solvent reorganization energy and entropy in hydrophobic hydration. J. Phys. Chem. B 104, 4964-4979.

Li, Z., and Lazaridis, T. (2012). Computing the thermodynamic contributions of interfacial water. Methods Mol. Biol. 819, 393-404.

Liu, P., Dehez, F., Cai, W.S., and Chipot, C. (2012). A Toolkit for the Analysis of Free-Energy Perturbation Calculations. J. Chem. Theory Comput. 8, 2606-2616.

Massova, I., and Kollman, P.A. (2000). Combined molecular mechanical and continuum solvent approach (MM-PBSA/GBSA) to predict ligand binding. Perspect. Drug Discovery Des. 18, 113-135.

Moustakas, D.T., Lang, P.T., Pegg, S., Pettersen, E., Kuntz, I.D., Brooijmans, N., and Rizzo, R.C. (2006). Development and validation of a modular, extensible docking program: DOCK 5. J. Comput.-Aided Mol. Des. 20, 601-619.

Nguyen, C.N., Young, T.K., and Gilson, M.K. (2012). Grid inhomogeneous solvation theory: Hydration structure and thermodynamics of the miniature receptor cucurbit[7]uril. J. Chem. Phys. 137, 044101.

Phillips, J.C., Braun, R., Wang, W., Gumbart, J., Tajkhorshid, E., Villa, E., Chipot, C., Skeel, R.D., Kale, L., and Schulten, K. (2005). Scalable molecular dynamics with NAMD. J. Comput. Chem. 26, 1781-1802.

Rao, F., and Caflisch, A. (2003). Replica exchange molecular dynamics simulations of reversible folding. The Journal of Chemical Physics 119, 4035.

Rush, T.S., Grant, J.A., Mosyak, L., and Nicholls, A. (2005). A shape-based 3-D scaffold hopping method and its application to a bacterial protein-protein interaction. J. Med. Chem. 48, 1489-1495.

Scior, T., Bender, A., Tresadern, G., Medina-Franco, J.L., Martínez-Mayorga, K., Langer, T., Cuanalo-Contreras, K., and Agrafiotis, D.K. (2012). Recognizing pitfalls in virtual screening: a critical review. J. Chem. Inf. Model. 52, 867-881.

Van Der Spoel, D., Lindahl, E., Hess, B., Groenhof, G., Mark, A.E., and Berendsen, H.J. (2005). GROMACS: fast, flexible, and free. J. Comput. Chem. 26, 1701-1718.

Verdonk, M.L., Cole, J.C., Hartshorn, M.J., Murray, C.W., and Taylor, R.D. (2003). Improved protein–ligand docking using GOLD. Proteins: Struct., Funct., Bioinf. 52, 609-623.

Young, T., Abel, R., Kim, B., Berne, B.J., and Friesner, R.A. (2007). Motifs for molecular recognition exploiting hydrophobic enclosure in protein-ligand binding. Proc. Natl. Acad. Sci. USA 104, 808-813.
