# Supplementary material for: Overcoming Chemical, Biological, and Computational Challenges in the Development of Inhibitors Targeting Protein-Protein Interactions
Source: Chem Biol. 2015 Jun 18;22(6):689–703. doi: 10.1016/j.chembiol.2015.04.019 (PMC4518475; doi:10.1016/j.chembiol.2015.04.019)
Supplement: Document S2. Article plus Supplemental Information [file mmc2.pdf]

Chemistry & Biology
Review

CellPress

Overcoming Chemical, Biological, and Computational Challenges in the Development of Inhibitors Targeting Protein-Protein Interactions

Luca Laraia,[1,2] Grahame McKenzie,[2] David R. Spring,[1] Ashok R. Venkitaraman,[2] and David J. Huggins[1,2,3,*]
[1]Department of Chemistry, University of Cambridge, Lensfield Road, Cambridge CB2 1EW, UK
[2]Medical Research Council Cancer Unit, University of Cambridge, Hutchison/MRC Research Centre, Hills Road, Cambridge CB2 0XZ, UK
[3]Theory of Condensed Matter Group, Cavendish Laboratory, University of Cambridge, 19 JJ Thomson Avenue, Cambridge CB3 0HE, UK
*Correspondence: djh210@cam.ac.uk
http://dx.doi.org/10.1016/j.chembiol.2015.04.019

Protein-protein interactions (PPIs) underlie the majority of biological processes, signaling, and disease. Approaches to modulate PPIs with small molecules have therefore attracted increasing interest over the past decade. However, there are a number of challenges inherent in developing small-molecule PPI inhibitors that have prevented these approaches from reaching their full potential. From target validation to small-molecule screening and lead optimization, identifying therapeutically relevant PPIs that can be successfully modulated by small molecules is not a simple task. Following the recent review by Arkin et al., which summarized the lessons learnt from prior successes, we focus in this article on the specific challenges of developing PPI inhibitors and detail the recent advances in chemistry, biology, and computation that facilitate overcoming them. We conclude by providing a perspective on the field and outlining four innovations that we see as key enabling steps for successful development of small-molecule inhibitors targeting PPIs.

Introduction

Protein-protein interactions (PPIs) have long been recognized as the key regulators of cellular pathways and networks. Developing tools to probe these interactions has led to an increased understanding of biological systems, and PPIs have also been targeted for drug development, due to the potential for selectively interfering with specific cellular pathways (Higueruelo et al., 2013; Mullard, 2012; Wells and McClendon, 2007). Indeed, several small-molecule modulators of PPIs are already in clinical use, while others are currently being evaluated in clinical trials (Table 1). A recent review focused on the properties of PPI inhibitors regarded as clinical success stories and discussed their specific mechanisms of action (Arkin et al., 2014). PPI inhibitors were separated into the classes of primary, secondary, and tertiary structural epitopes, as well as allosteric modulators. The future prospects for PPI-targeted drug discovery and the likelihood of success was discussed in each case. However, despite the notable successes, there have been many failures in identifying PPI inhibitors, and it is clear that inhibiting PPIs with small molecules remains a major challenge (Morelli et al., 2011; Villoutreix et al., 2014; Zinzalla and Thurston, 2009). In this review, we detail the specific chemical and biological challenges associated with inhibiting PPIs using small molecules, as well as the competitive advantages. We then discuss novel experimental and computational approaches to developing PPI inhibitors, with illustrative examples. A key point that we address concerns insights into the molecular basis for the reduced druggability of PPIs, in terms of how protein surfaces interact with small molecules. To focus on current approaches, we have chosen to cite recent applications of each approach rather than earlier work in their development.

Although most approved PPI inhibitors currently find application as treatments for cancer or in regulation of the immune system, therapeutics targeting infectious diseases such as HIV have also been approved. With a greater understanding of the cellular pathways in different organisms will come an increase in the ability of PPI inhibitors to target infectious diseases. At the same time, the availability of patient-specific and tumor-specific data from high-throughput genome sequencing will enhance the potential of PPI inhibitors for targeting cancer. Prior to the early 1990s, PPI inhibitors were primarily identified through phenotypic screening, consistent with drug discovery approaches at the time. From the more recent examples, it is interesting to note that clinical candidates were originally identified using a wide variety of different in vitro approaches, including radioligand binding assays, fluorescence-based assays, fragment-based drug discovery (FBDD), and peptide mimic approaches. This observation suggests that PPI drug targets should be approached using several experimental methods, to maximize the probability of finding promising small-molecule leads. Exploiting multiple approaches is important because different kinds of PPI exhibit significantly different structural characteristics and present different challenges. For example, inhibitors required to mimic linear protein sequences (such as integrin inhibitors) have proved more successful than inhibitors required to mimic single regions of secondary structure (such as α-helix or β-hairpin mimics), which in turn have proved more successful than inhibitors required to mimic discontinuous binding epitopes derived from tertiary structures (Arkin et al., 2014). In addition to small molecules, there has been great interest in the use of biologics to target PPIs. It is our opinion that, in the majority of cases, extracellular targets are best approached with biologics such as antibodies or protein drugs. In contrast, biologics are inherently less suitable for intracellular targets in the current state of the art, necessitating the use of small molecules. While the use of biologics to target PPIs is an interesting topic, we have chosen

CrossMark

Table 1. Examples of Small-Molecule PPI Modulators in Clinical Use or Currently Undergoing Clinical Trials, Including their Mode of Action, Identification Method, and Clinical Status

Name	Structure	Mode of Action	Identification Method	Clinical Status
Colchicine (Ahern et al., 1987)	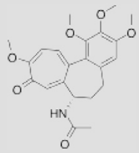	microtubule polymerization inhibitor	phenotypic screen	approved for gout
Vinblastine (Noble et al., 1977)	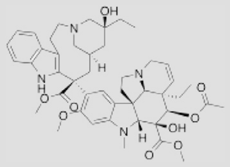	microtubule polymerization inhibitor	phenotypic screen	approved for several carcinomas
SAR1118 (Zhong et al., 2012)	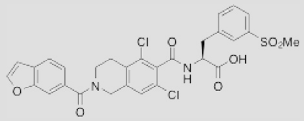	LFA-1/ICAM-1 inhibitor	peptide mimic	phase III for dry eye
Navitoclax (ABT-263) (Tse et al., 2008)	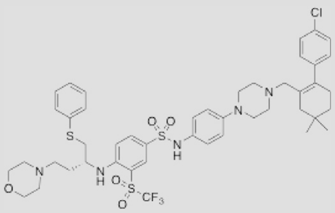	Bcl-2/Bcl-X_L inhibitor	fragment screen	phase II cancer
RG7112 (Vu et al., 2013)	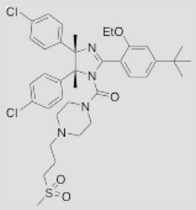	p53/MDM2 inhibitor	in vitro assay	phase Ib cancer
BI224436 (Fader et al., 2014)	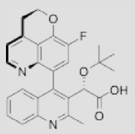	LEDGF/integrase inhibitor	in vitro assay	phase I HIV

LFA-1, lymphocyte function associated antigen 1; ICAM-1, intercellular adhesion molecule 1; Bcl-2, B-cell lymphoma 2; MDM2, mouse double minute 2; LEDGF, lens epithelium derived growth factor.

to limit the scope of this review to small molecules, peptides, and peptide mimics. For a thorough discussion on the subject of biologics, we refer the reader to other reviews (Leader et al., 2008; Sathish et al., 2013). However, it is worth nothing that many of the advantages and many of the challenges relevant to developing small-molecule inhibitors of PPIs are also relevant to the development of biologics.

PPI Inhibitors as Next-Generation Therapeutics

Expanding the Druggable Genome

The pharmaceutical industry has successfully developed drugs targeting only a small fraction of the components in the cellular signaling pathways that are misregulated in disease. Recent analysis of drug discovery efforts reveals that of the 15,000–20,000 genes encoded by the human genome, less than 300

 ©2015 Elsevier Ltd All rights reserved

have been specifically targeted with small molecules (Overington et al., 2006). More importantly, approximately two-thirds of these are directed against only ten classes of target, which comprise the so-called druggable genome. This analysis suggests that the size of the classically druggable genome is likely to be around 1,500 proteins at best. Expanding the druggable genome by accessing new target classes is therefore of the utmost importance in order to deliver improved health care. An accepted route to expanding this target repertoire is to generate molecules that inhibit the physical interaction of biological macromolecules (Archakov et al., 2003; Fry, 2006; Ruffner et al., 2007; Wells and McClendon, 2007). All cellular pathways are characterized by the physical interaction of biological macromolecules, most notably PPIs. Developing the technology required to find small-molecule inhibitors of PPIs represents a significant step toward expanding the druggable genome.

Increased Selectivity

Compared with the highly conserved nature of substrate binding pockets in enzyme classes such as kinases, PPI interfaces are inherently more diverse. For this reason, commentators have long postulated that PPI inhibitors will offer increased selectivity compared with existing small molecules. Maximizing target selectivity, of course, reduces the likelihood of off-target toxicities. In the well-publicized case of ATP-competitive kinase inhibitors, it is now an essential component of any assay cascade to rapidly ascertain the level of selectivity at target compared with the remainder of the kinome, by comparison against a panel of kinases (Davis et al., 2011). While multi-kinase inhibitors have shown clinical utility in some oncology settings (Rhodes et al., 2008), the need to reduce dose-limiting toxicities by increasing selectivity is a central driver for all drug discovery projects. For what reasons are PPI inhibitors likely to be more selective than their substrate-competitive counterparts? Perhaps the single most important reason is that the large surface area of a typical PPI interface offers more room to encode selectivity compared with the physically constrained environment of a substrate binding site. Incorporated within this concept is the fact that the chemical nature of substrate binding sites are defined absolutely by an invariant small molecule, while PPI interfaces have co-evolved together unhindered by a locked chemical structure, and, by definition, are therefore more diverse. At present, there is currently no PPI equivalent of a “kinase selectivity panel” other than the cell itself, and with only a small number of PPI inhibitors having made it to the clinic thus far, it is too early to draw any data-driven conclusions regarding this selectivity issue. However, the use of stapled α-helical peptides (see the section on Peptides and Peptide Mimics) provides encouraging preliminary data that selectivity at PPI interfaces is indeed achievable. Although still in its infancy, this approach has already generated numerous α-helical peptides that show clear target engagement and mechanism-dependent phenotypic responses in cell-based assays, and, more importantly, are tolerable and efficacious in in vivo models (Moellering et al., 2009). The importance of this observation lies in the fact that around 30% of all protein secondary structure is α-helical (Azzarito et al., 2013) and that, even with this limited template, specificity is achieved in the cell in order to drive orchestrated signaling pathways.

Reduced Susceptibility to Resistance Mutations

Clinical resistance to substrate-competitive enzyme inhibitors occurs through the selection of mutant enzymes in which inhibitor binding is prevented by the alteration of non-essential structural features that contribute to binding affinity, without affecting the ability of the enzyme to bind to its natural substrate. In contrast to the evolutionary conservation of enzyme active sites, the structural features that underlie PPIs are often quite distinct, even between closely related enzymes, in enabling their specific cellular functions. These diverse structural features would seem likely to make resistance mutations that decrease inhibitor binding without perturbing the natural substrate less frequent. Therefore, in principle, targeting the PPIs that underlie protein function offers an attractive alternative to active-site inhibition of enzymes. Combining ATP-competitive kinase inhibitors with allosteric inhibitors of the same target to stall or even prevent the emergence of resistance is a novel concept in cancer drug discovery. Recent studies from Novartis have supported this concept, at least in a pre-clinical setting (Adrián et al., 2006; Zhang et al., 2010). In these studies, the combination of the ATP-competitive Bcr-Abl inhibitor imatinib with an allosteric inhibitor, GNF-5, suppressed the emergence of resistance in cell culture experiments, and showed additive efficacy in an in vivo model of bone marrow transplantation. In cases where resistance mutations do occur, strategies to diminish their effect can be applied. These include machine-learning techniques, which have been applied to designing antimicrobial peptides (Fjell et al., 2009), and the substrate envelope hypothesis, whereby small-molecule inhibitors designed to mimic the shape of the natural substrates do not to lead to the development of resistance mutations (Parai et al., 2012).

Generating Novel Chemical Probes

In addition to widening the druggable genome and providing a wealth of new therapeutic targets, inhibitors of PPIs may also be useful chemical tools to probe cellular networks. Compared with small interfering RNA knockdown, they offer the potential of inhibiting a specific protein function without completely removing the protein from the cell. Several academic drug discovery platforms such as the NIH Molecular Libraries Program and the Structural Genomics Consortium (SGC) have made optimized compounds publicly available as probe compounds. In the case of the SGC, these follow stringent criteria making them suitable for effectively studying protein function: on-target potency must be better than 100 nM, selectivity must be at least 30-fold, while cellular potency must be better than 1 μM (http://www.thesgc.org/chemical-probes). Among these reported probes, PPI modulators of the BET bromodomains have been used to study their biological function and potential as anti-cancer therapeutics (Filippakopoulos et al., 2010).

Scope to Tailor Physical Properties

PPI inhibitors will by nature tend to be more solvent exposed than traditional active-site inhibitors because they bind at protein surfaces. While this is a disadvantage in terms of ligand efficiency (LE), it can be an advantage in terms of pharmacokinetic/pharmacodynamic (PK/PD) control. An inhibitor that is buried in a binding site is likely to have the majority of its surface in close contact with the protein and, thus, very little of its surface available for chemical elaboration. Conversely, a PPI inhibitor that is half exposed to solvent has a much greater scope for

chemical elaboration. This allows key physical properties such as the octanol/water partition coefficient (LogP) and polar surface area to be tailored without adversely affecting the binding affinity. This scenario has been exploited by Abbott for the development of Bcl-2 inhibitors in the progression from ABT-737 to ABT-263, where solvent-exposed positions were modified to optimize the balance between oral exposure in animals and efficacy in human tumor cell lines (Tse et al., 2008). A similar approach was adopted during the optimization of the MDM2/p53-inhibiting compounds, the Nutlins, to yield RG7112, an inhibitor now in human clinical trials (Vu et al., 2013). Increased solvent exposure of an inhibitor in complex also leads to more natural sites for synthetic coupling with cell-trafficking moieties such as peptides and sugars, in addition to other species appropriate for pro-drug strategies (Gynther et al., 2008, 2009).

Major Challenges of Developing PPI Inhibitors

Identifying Therapeutically Relevant PPIs

Therapeutic targets are either established on a case-by-case basis as a result of focused research efforts, often within academia, or are identified using unbiased screens that attempt to associate particular gene products with a specific cellular response or phenotype. In terms of focused research, recent efforts have been guided by studying PPI networks. Understanding such networks would allow for major advances in biology such as identifying synthetic lethal interactions, understanding modes of toxicity, and explaining the resilience of cellular networks to disruption (Hopkins, 2008); these are all important factors in drug discovery. In particular there has been a focus on understanding the role of hub proteins in cellular networks (Batada et al., 2006) and exploring their potential as drug targets (Hopkins, 2008). Computational work in this area holds much promise (Hood and Perlmutter, 2004; Yildirim et al., 2007), but the complexity of biological systems and the need to integrate diverse data and different methods means that successful application of systems biology to target selection remains a goal for the future.

RNAi has proved to be a successful tool for identifying new therapeutic targets that fall within the definition of classical drug targets, such as kinases, for both focused and unbiased approaches. However RNAi has, thus far, failed to expand the "druggable genome" beyond established target classes. This limitation can be explained simply by the fact that disruption of macromolecular assemblies by the loss of a single protein component is likely to lead to a confounded phenotypic effect, which is not directly attributable to the loss of that particular protein but to the perturbation of a higher-order macromolecular structure. In order to identify novel and therapeutically relevant PPIs, we propose that a different suite of target identification tools will be required. The most intuitive method for disrupting PPIs is to use ectopically expressed peptides to act in the manner of dominant negatives, thereby inhibiting PPIs. Screens of this type have been approached in a number of ways. In their simplest form, random peptide libraries can be generated and expressed in a mammalian cell line, and deflection from the intended phenotype measured using an appropriate assay. However, screening using random peptide libraries has been extensively investigated, and currently available methods suffer from low hit rates (as low as 1 in 10^6–10^7) that often preclude further progression (Roepe, 2001; Xu et al., 2001). New approaches addressing this problem remain a major unmet need.

Challenges of Druggability

The concept of druggability measures the suitability of a protein target or specific binding site for development of a small-molecule inhibitor. It is important to note that studying binding to the protein target in isolation does not consider PK/PD factors that influence druggability. Thus, bindability (Sheridan et al., 2010) or ligandability are perhaps better terms than druggability, but druggability tends to be the common parlance. The most obvious difficulty in targeting PPIs for drug development is the reduced druggability of protein surfaces in comparison with buried active sites, which have evolved to bind small molecules (Wells and McClendon, 2007). For example, in the work by Hajduk et al. (2005) on protein druggability of different protein classes derived from receptor-based nuclear magnetic resonance (NMR) screening of a fragment library, the protein binding targets are at the lower end of the druggability spectrum. Of the PPIs screened, only 30% were identified as containing a druggable binding site. This compares with the traditionally druggable protein kinases (45%), oxidoreductases (60%), and lyases (75%). In another study, druggability was correlated with compact pockets and rough surfaces, rather than the large and flat interfaces typically associated with PPIs (Wells and McClendon, 2007). The interaction energy between a ligand and a protein is derived from close contact between the two partners. In a buried binding site the protein surface can contact 100% of the ligand surface, whereas the interface may contact 50% or less of the ligand surface at a solvent-exposed protein surface. Thus, the inherent challenge in developing molecules that bind strongly to flat surfaces is the difficulty in achieving sufficient contact to yield the required interaction energy. For this reason, the expectation is that PPI inhibitors will need to be larger on average than traditional inhibitors to reach the same levels of potency. This assertion is supported by data from a number of studies (Higueruelo et al., 2009; Labbé et al., 2013; Morelli et al., 2011). For example, the PPI inhibitors studied in the TIMBAL database had a higher average molecular weight than drug-like molecules bound to proteins in the PDB (420 versus 360) and, in addition to being heavier, they also had a higher calculated octanol/water partition coefficient (cLogP) (4.0 versus 2.6). In terms of drug development, it is known that both molecular weight and lipophilicity are linked to poor PK/PD properties (Johnson et al., 2009). It is also interesting to consider the effect of these differences on the LE of PPI inhibitors. LE is a measure of the average contribution of each heavy atom to the binding affinity. It is commonly calculated from the using the pIC_{50} (logarithmic half-maximal inhibitory concentration) the compound and the number of heavy atoms it contains (HA), using Equation 1 (Hopkins et al., 2014).

$$LE = \frac{1.37 \times pIC_{50}}{HA} \qquad \text{(Equation 1)}$$

Lipophilic ligand efficiency (LLE) is another effective metric to drive decision making in medicinal chemistry, due to the deleterious effects of high lipophilicity on outcomes in drug discovery. LLE is calculated from the pIC_{50} of the compound and its cLogP,

 ©2015 Elsevier Ltd All rights reserved

CellPress

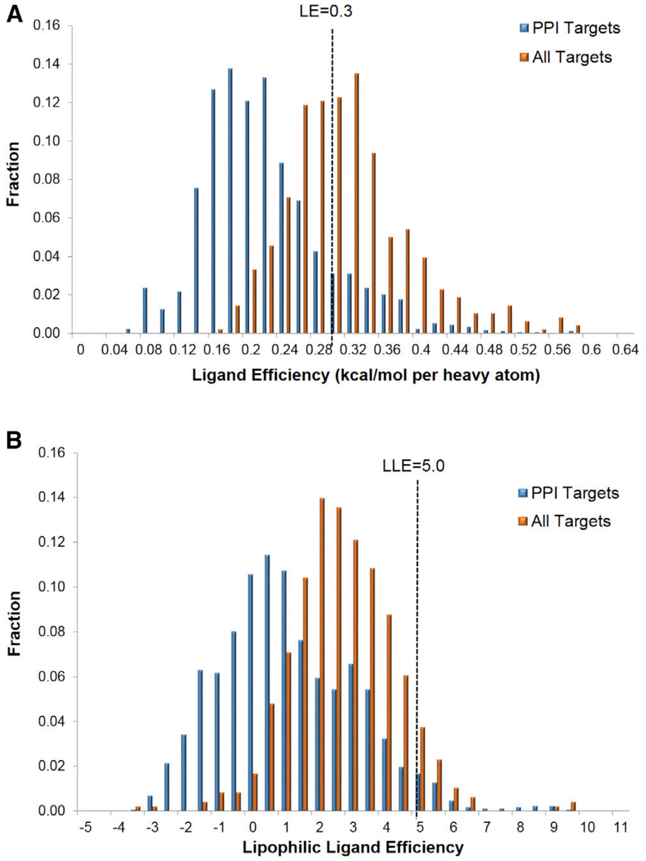

Figure 1. Distributions of Ligand Efficiency and Lipophilic Ligand Efficiency

Bar graphs showing the distributions of (A) ligand efficiency (LE) and (B) lipophilic ligand efficiency (LLE) using IC_{50} data for 1,736 small molecules in the TIMBAL database of PPI inhibitors and 37,143 small-molecule inhibitors in the curated portion of the BindingDB database. Heavy atom counts and cLogP values were computed using Schrödinger's Qikprop, and the small molecules were prepared using Schrödinger's Ligprep.

using Equation 2. It is sometimes termed lipophilic efficiency or LiPE.

$$LLE = pIC_{50} - cLogP \quad \text{(Equation 2)}$$

Figure 1 presents LE and LLE data for PPI inhibitors in the TIMBAL database and inhibitors in the BindingDB database (Liu et al., 2007). Integrins have been removed from the TIMBAL data due to difficulties in their curation. In addition, integrins are generally considered to have more in common with traditional drug targets than with PPI targets, as they tend to bind very short peptide motifs with high affinity. The results for the integrins are presented in Figure S1. The average LE for the PPI inhibitors studied was 0.23 kcal/mol per heavy atom, compared with an average of 0.32 kcal/mol per heavy atom for inhibitors in the BindingDB. The average LLE for the PPI inhibitors studied was 1.32, compared with an average of 3.12 for inhibitors in the BindingDB. As a guide the respective mean LE and LLE of oral drugs have been calculated as 0.45 kcal/mol per heavy atom and 4.43 (Gleeson et al., 2011; Hopkins et al., 2014), and it has been suggested that drug candidates should have an LE of greater than 0.30 kcal/mol per heavy atom (Hajduk, 2006; Hopkins et al., 2004) and an LLE of greater than 5.00 (Leeson and Springthorpe, 2007). Only 14.5% of the molecules in TIMBAL pass this LE filter and only 4.5% pass the LLE filter. Conversely, 54.8% of the molecules in the BindingDB pass the LE filter and 17.4% pass the LLE filter. In good agreement with this work, previous studies have calculated the average LE as 0.24 kcal/mol per heavy atom (Wells and McClendon, 2007) or 0.27 kcal/mol per heavy atom for PPI inhibitors and 0.32 kcal/mol per heavy atom for typical medicinal chemistry leads (Higueruelo et al., 2009). Based on these studies, a PPI inhibitor with 30 heavy atoms is expected to have a binding affinity of 1 μM, versus 90 nM for the typical medicinal chemistry lead (Hopkins et al., 2004). To reach 90 nM potency, the molecular weight of the PPI inhibitor would have to be increased by adding five to six heavy atoms. It is important to note that adding atoms to a ligand has a tendency to detrimentally affect its absorption, distribution, metabolism, and excretion (ADME) profile. Thus, PPIs typically have reduced druggability due to the inherent conflict between the two key goals of maximizing surface contact area and optimizing ADME properties. Reduced druggability will also have an impact upon high-throughput screening (HTS), as there will on average be fewer hits for a given library. This will lead to fewer alternative chemotypes available for drug development, and in some cases no viable hits. In particular, fragment screening against PPIs can be especially challenging (Dömling, 2008), unless the target has a strong binding hotspot or innovative strategies are applied (see the section on Fragment Screening). To address this problem, targeted small-molecule libraries for PPIs are now available, containing larger and more complex molecules (see the section on Customized HTS Libraries).

Structural Plasticity

While it is clear that PPIs can yield druggable targets, many of the success stories involve proteins that undergo structural changes upon binding (Aguirre et al., 2013). In general, these structural changes at the binding interface tend to reveal more lipophilic surfaces and pockets that complement lipophilic regions of the binding partner. Such protein flexibility will confound traditional structure-based approaches to target selection and lead optimization, because druggable pockets are not apparent in the apo protein structures. For example, proteins such as MDM2 are only predicted to be druggable when a liganded crystal structure is used for the analysis (Cheng et al., 2007). Figure 2 illustrates the extent of such structural changes in the case of six PPI inhibitors from the PDB (Berman et al., 2000). Figures 2A–2C illustrate inhibitors of Bcl-X_L, IL-2, and HDM2 overlaid on the protein structure from the apo state. The changes in protein structure mean that large portions of the inhibitors protrude into the protein surface. There are also cases, such as Keap1 (Figure 2D), where modest changes at the protein surface can increase the size of binding pockets and allow larger inhibitors than would be expected from an analysis of the apo structure. While there are cases where the apo and holo states are very similar, such as HIV integrase (Figure 2E) and KRas (Figure 2F), these seem to be in the minority. Six cases of proteins that undergo major structural changes upon binding are also presented in the review by Wells and McClendon (2007) on PPI inhibitors: IL-2, Bcl-X_L, HDM2, HPV11 × 10^2, ZipA, and TNF. The videos presented in their supporting information illustrate the remarkable extent of

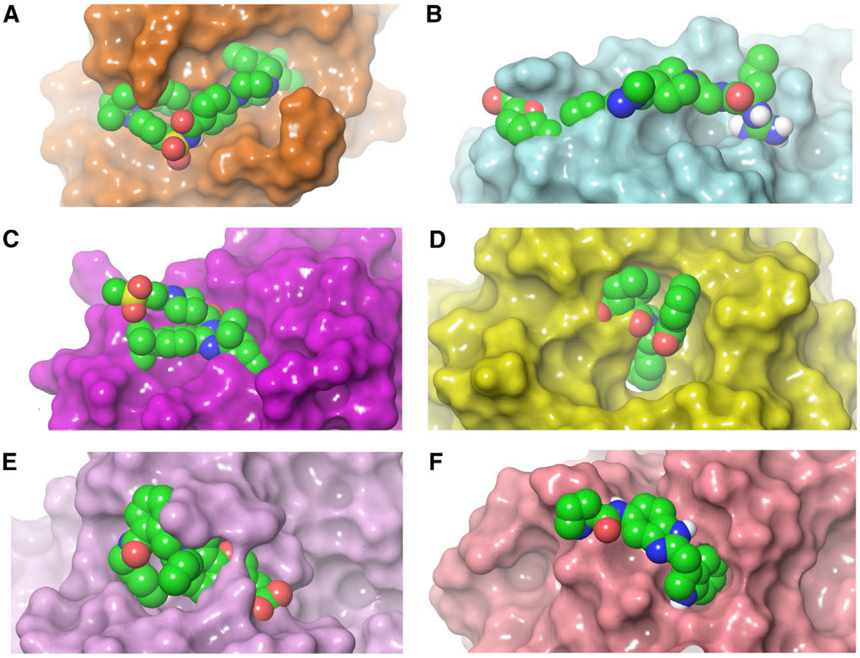

Figure 2. Apo Protein Structures of Six Surfaces Involved in PPIs, Showing Clashes with Ligands Overlaid from Protein-Ligand Complex Structures

The apo and holo structures were aligned using residues within 5.0 Å of the ligand, and the heavy atom root-mean-square deviation (RMSD) of these residues was calculated.

(A) Bcl-X_L from PDB: 1R2D overlaid with the ligand from PDB: 2O2N. The protein surface is shaded in orange and the RMSD is 1.51 Å.

(B) IL-2 from PDB: 1PY2 overlaid with the ligand from PDB: 3INK. The protein surface is shaded in cyan and the RMSD is 1.12 Å.

(C) HDM2 from PDB: 1Z1M overlaid with the ligand from PDB: 4IPF. The protein surface is shaded in magenta and the RMSD is 1.49 Å.

(D) Keap1 from PDB: 1ZGK overlaid with the ligand from PDB: 4IFN. The protein surface is shaded in yellow and the RMSD is 0.40 Å.

(E) HIV integrase from PDB: 1EX4 overlaid with the ligand from PDB: 4CE9. The protein surface is shaded in purple and the RMSD is 0.76 Å.

(F) KRas from PDB: 3GFT overlaid with the ligand from PDB: 4EPY. The protein surface is shaded in pink and the RMSD is 1.02 Å. The ligands are displayed using CPK atom coloring in all cases.

the structural changes between the complex with the native partner protein and the complex with the inhibitor.

Novel Experimental Tools for Targeting PPIs

Customized HTS Libraries

Interfaces between proteins are often large and lack a small-molecule active site; therefore, it is no surprise that reported inhibitors are distinct from traditional drugs. Recent analyses have shown that inhibitors of PPIs tend to be larger and more hydrophobic than traditional drugs or compounds found in current screening collections (see the section on Challenges of Druggability). These observations have led several groups to define rules to enrich screening collections for putative PPI inhibitors. Re-evaluating guidelines is certainly a prudent choice, given how poorly traditional compound collections have fared when screened against PPIs (Fry et al., 2013). Computational approaches to do this have recently been presented, and several new inhibitors of the p53/MDM2 interaction have been identified (Koes et al., 2012; Reynès et al., 2010). Another interesting development was the introduction of the ‘‘rule-of-four’’ (RO4), which states that compounds should have molecular weights of greater than 400, cLogP values of greater than 4, more than four rings, and more than four hydrogen-bond acceptors to deliver higher hit rates for PPIs (Morelli et al., 2011). However, one must note the increased risk of ADME failures in developing large, lipophilic compounds (see the section on Challenges of Druggability). In fact, recent data show that PPI inhibitors in clinical trials do not have higher cLogP values compared with non-PPI inhibitors despite having higher molecular weights (Kuenemann et al., 2014). Despite these caveats, the RO4 has subsequently been used to construct a PPI-focused library from commercially available compounds (Hamon et al., 2013). In addition to academic groups, companies have adopted such rules and have designed their own PPI-focused libraries. ChemDiv, Asinex, Comminex, Life Chemicals, Otava Chemicals, and NQuix all have libraries targeted at PPIs, which use both the RO4, decision trees, and machine-learning methods (Neugebauer et al., 2007), among other selection criteria (Hamon et al., 2013; Harris et al., 2011). A set of commercially available libraries is detailed in Table 2. Despite their promise, there are several more general limitations to current approaches to library design for PPIs (Laraia and Spring, 2013). The number of reported PPI inhibitors is relatively low, and the number of successfully inhibited targets is even lower; therefore, there are insufficient data for an accurate analysis to be conducted. In addition, very few PPI modulators have been approved for clinical use, and those that have are mostly natural products whose mechanism was only discovered subsequently. Examples include rapamycin, cyclosporine, and modulators of tubulin dynamics (Pommier and Marchand, 2012). Compounds used in the analyses are also heavily optimized and originate from both fragment and HTS approaches, and therefore do not necessarily reflect the requirements for an initial lead. The concept of a ‘‘lead-like’’ library has been around for many years (Teague et al., 1999), but a similar approach for PPIs has yet to be fully validated. The difficulty lies in the fact that PPIs are not a unified target class (like G-protein coupled receptors or kinases), which often contain structural similarities that can be leveraged in library design. Therefore, small-molecule inhibitors for different PPIs are unlikely to be similar to one another, except in their ability to bind to the hydrophobic patches at protein surfaces (Kuenemann et al., 2014). This would suggest that screening libraries should be as diverse as possible to cater for a variety of different PPIs (Huggins et al., 2011). The only exception to this may be PPIs mediated by secondary protein structures such as α helices (Whitby and Boger, 2012), which are discussed separately in the section on Peptides and Peptide Mimics. A validated approach for obtaining diverse screening collections is diversity-oriented synthesis (Galloway et al.,

 ©2015 Elsevier Ltd All rights reserved

Table 2. Commercial Libraries Targeted at PPIs

Supplier	No. of Compounds	Design Method	Website
Otava Chemicals	1,330	decision trees	http://www.otavachemicals.com/products/target-focused-libraries/protein-protein-interaction
Otava Chemicals	1,020	similarity search	http://www.otavachemicals.com/products/target-focused-libraries/protein-protein-interaction
Otava Chemicals	520	β-turn mimetics	http://www.otavachemicals.com/products/target-focused-libraries/peptidomimetic
Asinex	7,000	shape analysis	http://www.asinex.com/PPI_Library.html
ComInnex	custom	helix mimetics, macrocycles	http://www.cominnex.com/focused_and_targeted_libraries
Life Chemicals	850	machine learning	http://www.lifechemicals.com/services/targeted/general
Life Chemicals	23,200	2D fingerprint similarity	http://www.lifechemicals.com/services/targeted/general
Life Chemicals	4,300	rule-of-four	http://www.lifechemicals.com/services/targeted/general
NQuix	NA	NA	http://nquix.com/screening-libraries
ChemDiv	125,000	peptidomimetics	http://www.chemdiv.com/products/screening-libraries/chemdivs-screening-libraries-list/

2010), whose mantra is that compounds containing as many diverse scaffolds as possible are synthesized efficiently in few steps. Diversity is generally assessed using computational methods, and several PPI inhibitors have been identified using this approach (Marcaurelle et al., 2009). However, more research is required to fully evaluate the potential of such libraries for the identification of PPI inhibitors. With so many companies offering targeted PPI libraries, it should only be a matter of time before these approaches to library generation are validated or discredited.

Assay Platforms for HTS

Screening methods for inhibiting PPIs vary depending on the overall approach taken (Pagliaro et al., 2004). Different techniques can be used for fragment screening (see the section on Fragment Screening) rather than the more traditional HTS approaches (Winter et al., 2012). For HTS, the most simple and widely used approach is the fluorescence polarization (FP) assay (Arkin et al., 2004). This technique requires that one component of the PPI can be truncated to a smaller peptide that still retains affinity for the other protein. This peptide is then attached to a fluorophore, and the change in tumbling rate between bound and unbound states forms the basis for the assay window. These assays are fast to run, amenable to 384- or even 1,536-well formats, and require very little labeled peptide and protein. This makes them ideal for HTS, which is why they have been used on a wide scale. One of the downsides to this system is the requirement for one of the two interacting proteins to be truncated to a small (<40 amino acids) peptide. While FP is a suitable technique for many PPIs, it may not be appropriate for PPIs with extremely large binding interfaces or those with discontinuous binding epitopes. In this case, ELISAs may offer an alternative, as two full-length proteins can be used. However, the throughput of ELISA assays is significantly lower. Other widely used assay techniques include Förster Resonance Energy Transfer (FRET) and AlphaScreen, both of which are extensively discussed elsewhere (Arkin et al., 2004). An important issue with these high-throughput assay techniques is the high rate of false positives, which can occur as a result of fluorescent molecules that interfere with the assay, as well as redox-active compounds or protein-precipitating compounds. Ideally such compounds would already be removed during the preparation of the screening library, and data-driven computational filters are already available for this purpose (Baell and Holloway, 2010). However, if compounds that do not pass these filters are included in screening libraries, close attention must be paid to the results from follow-up assays. Biophysical techniques (Dias and Ciulli, 2014; Pfaff et al., 2015) have been used for this purpose and include NMR, surface plasmon resonance, and isothermal titration calorimetry. X-Ray crystallography offers a final validation of binding and allows structure-based drug design. All recent successful PPI projects have benefited from structural information, as rearrangements in the protein upon small-molecule binding are frequently observed (see the section on Structural Plasticity). One could argue that traditional HTS, in addition to fragment screening efforts against PPIs, would struggle enormously without structural data.

Fragment Screening

FBDD has been adapted on a wide scale in the last 10–15 years. One of the key advantages of this approach is the more efficient coverage of chemical space by compounds of lower molecular weight, requiring smaller screening libraries compared with HTS. In the context of PPIs, numerous examples of inhibitors have been identified using this approach (Abdel-Rahman et al., 2011; Douse et al., 2015; Gao et al., 2014; Holvey et al., 2015; Jose et al., 2012; Lund et al., 2015; Molzan et al., 2012; Moore et al., 2009; Patrone et al., 2013; Van Molle et al., 2012; Yin et al., 2014). Prominent examples include the Bcl-2 inhibitor ABT-737 (Oltersdorf et al., 2005), inhibitors of the RAS oncogene (Maurer et al., 2012), and inhibitors of the BET bromodomains (Chung et al., 2012). As PPIs tend to stretch over a large surface area but contain hotspots contributing a large proportion of the binding energy, fragments would appear suitable to identify these sites (Coyne et al., 2010; Scott et al., 2013; Valkov et al., 2012). However, the risk of missing fragments whose binding affinity is beyond the limits of detection exists. This is more likely to be the case in PPIs, where a fragment will cover a smaller area of the overall binding interface. An interesting approach to partially circumvent this problem is the use of tethering fragments (Wilson

and Arkin, 2013). These contain a thiol moiety for crosslinking with protein thiols before engaging in non-covalent interactions. This allows the adduct to be detected by mass spectrometry, and was successfully used to identify an allosteric site to the interleukin/interleukin-receptor binding interface (Braisted et al., 2003). A criticism of the FBDD has been the lack of three-dimensionality in the screening collections (Morley et al., 2013). To address this issue, researchers at the Broad Institute have implemented a diversity-oriented synthesis approach to design sp^3 and stereochemically rich fragments (Hung et al., 2011). The resulting effect on rate and quality of hits will determine the utility of these compounds. In addition to providing valuable hits for further elaboration, fragment screening has gained traction as a tool to establish PPI druggability (Edfeldt et al., 2011). It has been hypothesized that low hit rates in fragment screens occur as a result of poor druggability, and that therefore in such cases it may be wise to re-assess screening programs of any kind for a given target or to select other approaches such as rational design. In summary, FBDD is a welcome addition to the repertoire of techniques available for identifying PPI modulators, and we believe that it complements rather than replaces existing strategies such as HTS. If resources are available and the target sufficiently validated, we would advocate the use of such approaches in parallel to maximize the chances of success.

Peptides and Peptide Mimics

Several research groups have shown that a large percentage of PPIs are mediated by protein secondary structures. In particular, α helices occur frequently on protein interfaces, and short α-helical peptides based on the key binding hotspot may provide suitable inhibitors of PPIs (Bullock et al., 2011). This enticing hypothesis would suggest that every α-helix-containing PPI may have a ''ready-made'' lead compound available. However, unmodified peptides tend to be poor inhibitors due to the large entropic penalty of binding to their target. Therefore, the search for stabilized peptides and small molecules that mimic protein secondary structure has recently been an active area of research (Azzarito et al., 2013; Lao et al., 2014; Lee et al., 2011; Walensky and Bird, 2014). Pioneering work by Grubbs, Verdine, Walensky, and Sawyer on cyclizing alkene-containing peptides using ring-closing metathesis has been shown to increase α helicity, potency, and stability for select examples (Blackwell and Grubbs, 1998; Chang et al., 2013; Moellering et al., 2009). Aileron, a company founded to pursue this approach, has successfully completed its first phase I clinical trial targeting growth hormone-releasing hormone (Grigoryev, 2013). Other criticisms of peptide therapeutics are the lack of cell permeability in the absence of a specific targeting sequence and poor PK/PD properties. The success of the stapled peptide approach has been the ability to obtain cell-permeable, active peptides without tags and with improved PK/PD properties. However, this success was achieved through trial and error rather than rational design, because permeability and stability are very difficult to calculate a priori. Thus, it is necessary to conduct extensive peptide structure-activity relationships (SAR) to identify suitable candidates. Despite this, stapled or cyclic peptides remain an exciting approach to tackle PPIs, especially if one considers that several naturally occurring cyclic peptides are already approved drugs with acceptable properties (Liskamp et al., 2008). Different methods of cyclization and stabilization have now been reported, providing a wealth of options to those attempting this approach (Lau et al., 2014).

In addition to stabilized peptides, small-molecule scaffolds that mimic protein secondary structure have been reported by several groups. Pioneering work by Hamilton identified the terphenyl scaffold as an α-helix surrogate (Cummings and Hamilton, 2010). Second-generation scaffolds that possess improved solubility and synthetic tractability have been identified (Shaginian et al., 2009). Of particular note is the work by the Wilson group on the solid-phase synthesis of oligo-amides (Murphy et al., 2013). This mimic can now be assembled rapidly with simple preparation and purification methods, and different building blocks are now available for every amino acid. Despite promising work in the field, potent inhibitors derived from this approach have yet to be published, and it seems increasingly unlikely that a singular scaffold will be applicable to all α-helix-mediated PPIs. However, it continues to be a fruitful approach for discovering probe compounds, and further developments may provide compounds for clinical testing.

Novel Computational Tools for Targeting PPIs

In this section we discuss the computational tools that have been used to facilitate and understand PPIs and to aid in the discovery of inhibitors. A number of the computational techniques discussed in this article are described in Table S1, along with references to available software and theory papers.

Predicting PPI Interfaces

Studying a PPI as a potential therapeutic target first requires identification and characterization of the binding interface. A number of computational methods have been used to identify PPI interfaces from protein structures (Fernández-Recio, 2011; Fuller et al., 2009). Computational predictions based on a consensus neural network method were found to yield 80% prediction accuracy with 51% coverage on a set of 100 non-homologous protein chains taken from PPI complexes (Chen and Zhou, 2005). Statistical methods based on residue pair frequencies (Negi and Braun, 2007), frequencies of short polypeptide sequences (Pitre et al., 2006), and probabilistic analysis of orthogonal protein features show similar predictive power (Scott and Barton, 2007). Methods based on the similarity of interface regions have also been successfully employed to predict the structure of binding interfaces, but require a template in the reference dataset to achieve this (Tuncbag et al., 2012). However, due to the effect of protein flexibility discussed above, it is easier to identify a binding interface from a separated PPI complex, and a generally applicable method should be able to identify the binding interface from protein structures in their separated structures. It is interesting to note that the abilities of numerous methods to achieve this task have been independently assessed by CAPRI (Critical Assessment of PRedicted Interactions), an ongoing experiment to assess the ability of protein-docking methods to predict PPIs. Through 9 years and four rounds of testing on 42 test cases, the conclusion is that current methods yield reasonably accurate models, but only in the absence of major conformational changes (Janin, 2010). In addition to identifying binding interfaces, it would be very useful to have the power to identify potential allosteric binding sites that modulate PPIs (Freire, 2000). While efforts have been focused

 ©2015 Elsevier Ltd All rights reserved

in this direction (Demerdash et al., 2009), it remains an area in need of significant improvement.

Identifying Binding Hotspots

Hotspots were originally identified as residues at a PPI interface that contribute significantly to the binding affinity, such that their mutation to alanine leads to a significant reduction in binding affinity (Bogan and Thorn, 1998; Clackson and Wells, 1995). The term has also been used to refer to clusters of such residues, which we refer to here as protein surface hotspots. The term hotspot has also been identified with a site on a protein that has high propensity for ligand binding, and we refer to these sites as ligand binding hotspots. The determinants that underlie both phenomena are very similar, and here we use the term hotspot to overarch the two (Zerbe et al., 2012). There are a number of methods for identifying hotspot regions at protein surfaces (Villoutreix et al., 2014), commonly assessed by their ability to recapitulate experimental data from sources such as the ASEdb alanine scanning energetics database (Thorn and Bogan, 2001), the BID (Binding Interface Database) (Fischer et al., 2003), and the HotSprint database (Guney et al., 2008). The first class of methods is empirical and correlates experimental data with surface properties such as protein curvature, electrostatic potential, or hydrophobicity. This approach has generated software that accurately predicts protein surface hotspots, such as HotPoint (Tuncbag et al., 2009), and software that accurately predicts ligand binding hotspots, such as SiteMap (Halgren, 2009). The second class of methods uses explicit computational alanine scanning, which predicts changes in binding free energy upon mutation to alanine. This can be achieved by free energy methods such as molecular mechanics/generalized Born surface area (Gohlke et al., 2003), free energy perturbation (FEP), and thermodynamic integration (TI) (Moreira et al., 2007). Recent studies suggest that the MM-GBSA method yields accurate results that are comparable or better than more computationally intensive TI calculations (Martins et al., 2013). Other studies suggest that Poisson Boltzmann implicit solvation is more accurate than generalized Born implicit solvation in the context of computational alanine scanning (Bradshaw et al., 2011).

The third class of methods involves physics-based analysis of ligand binding hotspots. This includes analysis of probe fragments (Brenke et al., 2009), which suggest where larger ligands will bind, but also water molecules (Haider and Huggins, 2013). Hydrophobic desolvation is a key driver of PPIs, and binding hotspots are often found in hydrophobic regions. For this reason, it is useful to consider water at PPI interfaces and also its displacement by other small molecules (Landon et al., 2007). Solvation has also been explicitly modeled using FEP, TI, and inhomogeneous fluid solvation theory (IFST) (Huggins and Payne, 2013; Li and Lazaridis, 2006). Schrodinger's WaterMap is a commercially available IFST software package that is widely used in the pharmaceutical industry to understand SAR, and has been used to understand the determinants of affinity in the PPIs of PLK1 (Huggins et al., 2010) and identify ligand binding hotspots on the FKBP12 protein (Beuming et al., 2012). In this study, predicted hotspots correlate positively with a high hit rate in NMR screening of fragments. A number of web server tools for hotspot prediction are now available in all three classes, including Robetta (Kim et al., 2004), DrugScore^{PPI} (Krüger and Gohlke, 2010), and HotPoint (Tuncbag et al., 2010). A list of such tools can be found at http://www.vls3d.com. Within any of these approaches, it is clear that protein flexibility must be modeled to yield a generally applicable tool for identifying binding hotspots (Lexa and Carlson, 2010).

Modeling Molecular Flexibility

As discussed above, protein flexibility is a very important feature of molecular recognition for PPIs (Brown and Hajduk, 2006). This is true for interactions between native partners and for small-molecule inhibitors. This means that conformational flexibility must be considered explicitly for computational methods to be effective in modeling a broad range of PPIs (see the section on Structural Plasticity). One approach that has proved useful in molecular docking is the use of predefined structural ensembles. In this case, an ensemble of multiple protein structures is used for analysis rather than one single protein structure. A recent review notes that it leads to better performance than the worst single protein structure in almost all cases (Korb et al., 2012). Thus, approaches based on structural ensembles are preferred because the virtual screening performance of a single protein structure for a given ligand is unknown. In terms of selecting the ensemble, protein structures can be derived from experimental techniques such as NMR and X-ray crystallography (Damm and Carlson, 2007) or from computational techniques such as molecular dynamics (MD) (Cheng et al., 2008). Crucially, it is clear that the selection of the ensemble is a critical determinant of performance for molecular docking (Korb et al., 2012), druggability assessments (Brown and Hajduk, 2006), and hotspot identification (Metz et al., 2012). While there have been advances in modeling induced fit effects, major difficulties remain in modeling major domain motions (Wells and McClendon, 2007). The two key aspects of effective computational modeling are comprehensive sampling techniques and accurate estimation of free energy. Modeling large domain motion requires both of these aspects. Thus, accurately computing the energetic cost of protein rearrangement is achievable using FEP methods, but only in cases where the ligand binding mode is known (Wang et al., 2013). Similarly, replica exchange MD (REMD) (Miyashita et al., 2009) and enveloping distribution sampling (Riniker et al., 2011) have both been successfully applied to model large domain motion, but extending these methods to virtual screening and combinatorial molecular design is beyond the scope of current computational power. However, computational methods have shown promise in identifying transient pockets at PPIs. These cryptic pockets are not present in the apo structure of the protein, but are revealed upon ligand binding. Importantly, such pockets are not an uncommon feature (Bernini et al., 2014; Foster et al., 2012; Schames et al., 2004; Tan et al., 2012) and are promising targets for therapeutic intervention (Johnson and Karanicolas, 2013). Because these pockets are not present in the majority of structures that make up the conformational ensemble in the apo state, they can be difficult to identify using conventional MD simulations. For this reason, methods that are based on probe molecules, such as MixMD (Lexa and Carlson, 2013) and SILCS (Foster et al., 2012; Raman et al., 2011), have proved more effective. One additional factor is the interplay of the degrees of freedom for the water and protein, which can lead to enthalpy/entropy compensation and confound commonly applied computational approaches (Breiten et al., 2013). This is

 ©2015 Elsevier Ltd All rights reserved

an area where progress is needed to improve the predictive power of computational methods.

Virtual Screening

Virtual screening (VS) is often used in an attempt to enrich compound libraries for molecules with an increased likelihood of hitting a particular target. The two main methods used for VS are structure-based and ligand-based screening (Ripphausen et al., 2010). Structure-based screening is commonly performed using one of three techniques. Molecular docking uses an atomistic description to compute the ligand-protein interactions, pharmacophore screening matches the features of the ligand to those of the binding site, and shape-based screening assesses the geometric fit between the ligand and the binding site. Ligand-based screening is used to identify new hit molecules using information about existing hit molecules. There are a number of pitfalls associated with the use of VS (Scior et al., 2012), and these should be understood before applying it. It can also be useful to utilize an ensemble of protein structures (see the section on Modeling Molecular Flexibility) in all these approaches (Fan et al., 2009; Totrov and Abagyan, 2008). One might expect that VS would find greater utility in identifying PPI inhibitors, due to the lower experimental hit rates and requirement to test larger and more complex molecules. However, the majority of VS methods have been optimized for buried active sites, and it is not clear that these will translate to calculations at protein surfaces. Despite this, existing and purpose-built VS approaches have shown promise (Fernandez-Recio et al., 2004; Rouhana et al., 2013; Villoutreix et al., 2014). For example, a "fuzzy" pharmacophore model combined with GOLD docking (Jones et al., 1995) was used to identify interferon-α inhibitors (Geppert et al., 2012), and consensus scoring using DOCK4 (Ewing and Kuntz, 1997) was used to identify STAT3 inhibitors (Matsuno et al., 2010). In addition, pharmacophore tools based on key anchor residues between PPI partners has been used to identify inhibitors of the p53-MDM2 interaction (Koes et al., 2012). Utilizing information from native interactions is likely to be a key enabling step in the efficient design of PPI inhibitors.

Conclusions and Future Perspectives

There are a number of reasons why developing therapeutics to target PPIs is a challenging process. In general, high-affinity protein-ligand binding is a driver of drug efficacy and is one of the key goals in early-stage drug discovery. This high-affinity binding is derived from close contact at the protein-ligand interface, and at protein surfaces a significant proportion of the ligand is exposed to solvent rather than in contact with the protein. Thus, for a given level of binding affinity, PPI inhibitors tend to be larger than inhibitors of buried binding sites. An increase in size brings with it a greater risk of PK/PD liabilities that may lead to drug failure. For this reason, PPI targets are considered to be inherently less druggable than traditional targets. Additional difficulties arise from the confounding effect of surface flexibility on structure-based drug design, and the challenges of target selection due to the complexity of cellular networks. However, PPI inhibitors hold great promise for the generation of selective therapeutics for a variety of diseases if these difficulties can be overcome. For this reason, great efforts have been focused on devising novel chemical, biological, and computational tools to aid in the process of developing PPI inhibitors. These tools are described in this review. In the future, we see four key areas where advances in our understanding and increases in the utility of computational techniques will further the development of PPI inhibitors.

To date, PPIs have been targeted only sporadically with small molecules, at least partly because existing RNAi technologies are unable to associate specific PPIs with specific cellular phenotypes. Indeed, knockdown of candidate targets with RNAi can often be uninformative, due to the simultaneous depletion of beneficial as well as disease-associated protein interfaces. Therefore, since the current state of the art for target identification and validation is unsuitable for the identification of protein interfaces, it is not surprising that few PPIs have been validated as prospective targets using current tools. The problem of target selection is an area where computational approaches to systems biology hold great promise (Kreeger and Lauffenburger, 2010). However, it will be vital to use computational models to design experiments that are able to verify which protein targets within a cellular network are most amenable to selective interference to achieve the desired goal. Molecular biology is now an immensely powerful field, but probing a complex system requires careful study. This is an area where academic work can contribute significantly to industrial progress. Understanding particular cellular pathways and the PPIs involved can take many years of work, but is a fruitful field for publication during this time and can be exploited at the conclusion for commercial purposes. The development of methods for the high-throughput identification of druggable PPIs for a given pathway or phenotype would significantly expedite the process of drug discovery against PPIs.

Academic research can also prove useful in the related process of validating difficult drug targets such as PPIs. Pharmaceutical companies are naturally wary of the risks associated with developing PPI inhibitors, and this is particularly true for unvalidated protein targets. The process of target validation can be a lengthy process and requires a coordinated multidisciplinary approach. For this reason, large initiatives such as the NIH Accelerating Medicines Partnership, the Wellcome Trust Seeding Drug Discovery funding, and the UK Technology Strategy Board Biomedical Catalyst funding will be a key part of target validation in the future. Publicly available data on the therapeutic potential of targeting all relevant proteins in the human genome would greatly enhance decision-making processes. However, the breadth and heterogeneity of genetic data will require cleverly designed and well-maintained databases.

Publicly available data will also enable the design of effective screening libraries for PPIs. The results of many PPI screens with many libraries, including those where no leads were ultimately identified, will provide valuable information on whether particular libraries will fare well against PPIs, and whether particular PPIs may not be amenable to small-molecule inhibition. Unfortunately, both academia and industry are reluctant to publish negative results, and positive results are often delayed due to patent issues. Even when screens with positive results are published, a reader will rarely have access to all compound structures and associated activities. Only with a complete dataset can a comprehensive analysis be carried out. We envisage that the current drive for "big data" will help to separate druggable and undruggable PPIs and validate effective screening libraries for PPIs.

 ©2015 Elsevier Ltd All rights reserved

One area in the development of PPI inhibitors where experimental data may not prove as fruitful is the identification of cryptic binding pockets at protein surfaces. Such pockets are often druggable but are not identified by X-ray crystallography of the apo protein structure. Brute force experimental approaches using HTS or arrayed library synthesis can work but the vast size of chemical space means that such approaches will commonly fail. Conversely, computational methods can search the conformational space of the protein surface and identify the presence or absence of druggable pockets. The two barriers to achieving this are the two main issues that have always existed in computational drug discovery: sampling and scoring (Schneider, 2012). Significant progress has already been made in circumventing the first of these hurdles, using enhanced sampling such as REMD and long-timescale calculations with multiple processors. It is the second barrier that is the current challenge, with many classical force fields failing to generate accurate protein-structural ensembles (Beauchamp et al., 2012) and quantum mechanical approaches still too computationally expensive for the analysis of such large systems. Further increases in computing power will allow better models to be applied to larger systems, and allow druggable cryptic binding pockets to be identified from crystallographic apo structures. These approaches will also improve our understanding of allosteric modulation of PPIs.

In summary, the development of effective therapeutics from PPI inhibitors will be improved by the widespread dissemination of relevant data from large multidisciplinary projects, the effective use of such data, and the exploitation of increased computing power to accurately model ensembles of protein structures. Science is already moving in these directions, but academia and industry will need to work together in order to turn this movement into positive outcomes for society.

SUPPLEMENTAL INFORMATION

Supplemental Information includes one figure and one table and can be found with this article online at http://dx.doi.org/10.1016/j.chembiol.2015.04.019.

ACKNOWLEDGMENTS

Work in the D.R.S. laboratory is supported by the European Union, Engineering and Physical Sciences Research Council, Biotechnology and Biological Sciences Research Council, Medical Research Council, and Wellcome Trust. Work in the A.R.V. laboratory is supported by the Medical Research Council and Wellcome Trust. Work in the D.J.H. laboratory is supported by the Medical Research Council under grant ML/L007266/1. All calculations were performed using the Darwin Supercomputer of the University of Cambridge High Performance Computing Service (http://www.hpc.cam.ac.uk/) provided by Dell Inc. using Strategic Research Infrastructure Funding from the Higher Education Funding Council for England, and were funded by the EPSRC under grants EP/F032773/1 and EP/J017639/1. G.J.M. and A.R.V. are affiliated with Phore-Most Ltd, Cambridge. We thank Alicia Higueruelo and John Skidmore for helpful discussions.

REFERENCES

Abdel-Rahman, N., Martinez-Arias, A., and Blundell, T.L. (2011). Probing the druggability of protein-protein interactions: targeting the Notch1 receptor ankyrin domain using a fragment-based approach. Biochem. Soc. Trans. *39*, 1327–1333.

Adrián, F.J., Ding, Q., Sim, T., Velentza, A., Sloan, C., Liu, Y., Zhang, G., Hur, W., Ding, S., and Manley, P. (2006). Allosteric inhibitors of Bcr-abl-dependent cell proliferation. Nat. Chem. Biol. *2*, 95–102.

Aguirre, C., Ten Brink, T., Walker, O., Guilliere, F., Davesne, D., and Krimm, I. (2013). BcL-xL conformational changes upon fragment binding revealed by NMR. PLoS One *8*, e64400.

Ahern, M., Reid, C., Gordon, T., McCredie, M., Brooks, P., and Jones, M. (1987). Does colchicine work? The results of the first controlled study in acute gout. Aust. N. Z. J. Med. *17*, 301–304.

Archakov, A.I., Govorun, V.M., Dubanov, A.V., Ivanov, Y.D., Veselovsky, A.V., Lewi, P., and Janssen, P. (2003). Protein-protein interactions as a target for drugs in proteomics. Proteomics *3*, 380–391.

Arkin, M., Glicksman, M., Fu, H., Havel, J., and Du, Y. (2004). Inhibition of Protein-Protein Interactions: Non-cellular Assay Formats (Eli Lilly and the National Center for Advancing Translational Sciences).

Arkin, M.R., Tang, Y., and Wells, J.A. (2014). Small-molecule inhibitors of protein-protein interactions: progressing toward the reality. Chem. Biol. *21*, 1102–1114.

Azzarito, V., Long, K., Murphy, N.S., and Wilson, A.J. (2013). Inhibition of [alpha]-helix-mediated protein-protein interactions using designed molecules. Nat. Chem. *5*, 161–173.

Baell, J.B., and Holloway, G.A. (2010). New substructure filters for removal of pan assay interference compounds (PAINS) from screening libraries and for their exclusion in bioassays. J. Med. Chem. *53*, 2719–2740.

Batada, N.N., Hurst, L.D., and Tyers, M. (2006). Evolutionary and physiological importance of hub proteins. PLoS Comput. Biol. *2*, e88.

Beauchamp, K.A., Lin, Y.-S., Das, R., and Pande, V.S. (2012). Are protein force fields getting better? A systematic benchmark on 524 diverse NMR measurements. J. Chem. Theor. Comput. *8*, 1409–1414.

Berman, H.M., Westbrook, J., Feng, Z., Gilliland, G., Bhat, T.N., Weissig, H., Shindyalov, I.N., and Bourne, P.E. (2000). The Protein Data Bank. Nucleic Acids Res. *28*, 235–242.

Bernini, A., Henrici De Angelis, L., Morandi, E., Spiga, O., Santucci, A., Assfalg, M., Molinari, H., Pillozzi, S., Arcangeli, A., and Niccolai, N. (2014). Searching for protein binding sites from molecular dynamics simulations and paramagnetic fragment-based NMR studies. Biochim. Biophys. Acta *1844*, 561–566.

Beuming, T., Che, Y., Abel, R., Kim, B., Shanmugasundaram, V., and Sherman, W. (2012). Thermodynamic analysis of water molecules at the surface of proteins and applications to binding site prediction and characterization. Proteins *80*, 871–883.

Blackwell, H.E., and Grubbs, R.H. (1998). Highly efficient synthesis of covalently cross-linked peptide helices by ring-closing metathesis. Angew. Chem. Int. Ed. Engl. *37*, 3281–3284.

Bogan, A.A., and Thorn, K.S. (1998). Anatomy of hot spots in protein interfaces. J. Mol. Biol. *280*, 1–9.

Bradshaw, R.T., Patel, B.H., Tate, E.W., Leatherbarrow, R.J., and Gould, I.R. (2011). Comparing experimental and computational alanine scanning techniques for probing a prototypical protein–protein interaction. Protein Eng. Des. Sel. *24*, 197–207.

Braisted, A.C., Oslob, J.D., Delano, W.L., Hyde, J., McDowell, R.S., Waal, N., Yu, C., Arkin, M.R., and Raimundo, B.C. (2003). Discovery of a potent small molecule IL-2 inhibitor through fragment assembly. J. Am. Chem. Soc. *125*, 3714–3715.

Breiten, B., Lockett, M.R., Sherman, W., Fujita, S., Al-Sayah, M.H., Lange, H., Bowers, C.M., Heroux, A., Krilov, G., and Whitesides, G.M. (2013). Water networks contribute to enthalpy/entropy compensation in protein-ligand binding. J. Am. Chem. Soc. *135*, 15579–15584.

Brenke, R., Kozakov, D., Chuang, G.-Y., Beglov, D., Hall, D., Landon, M.R., Mattos, C., and Vajda, S. (2009). Fragment-based identification of druggable 'hot spots' of proteins using Fourier domain correlation techniques. Bioinformatics *25*, 621–627.

Brown, S.P., and Hajduk, P.J. (2006). Effects of conformational dynamics on predicted protein druggability. ChemMedChem *1*, 70–72.

Bullock, B.N., Jochim, A.L., and Arora, P.S. (2011). Assessing helical protein interfaces for inhibitor design. J. Am. Chem. Soc. *133*, 14220–14223.

 ©2015 Elsevier Ltd All rights reserved

Chang, Y.S., Graves, B., Guerlavais, V., Tovar, C., Packman, K., To, K.-H., Olson, K.A., Kesavan, K., Gangurde, P., Mukherjee, A., et al. (2013). Stapled α-helical peptide drug development: a potent dual inhibitor of MDM2 and MDMX for p53-dependent cancer therapy. Proc. Natl. Acad. Sci. USA *110*, E3445–E3454.

Chen, H., and Zhou, H.X. (2005). Prediction of interface residues in protein–protein complexes by a consensus neural network method: test against NMR data. Proteins *61*, 21–35.

Cheng, A.C., Coleman, R.G., Smyth, K.T., Cao, Q., Soulard, P., Caffrey, D.R., Salzberg, A.C., and Huang, E.S. (2007). Structure-based maximal affinity model predicts small-molecule druggability. Nat. Biotechnol. *25*, 71–75.

Cheng, L.S., Amaro, R.E., Xu, D., Li, W.W., Arzberger, P.W., and McCammon, J.A. (2008). Ensemble-based virtual screening reveals potential novel antiviral compounds for avian influenza neuraminidase. J. Med. Chem. *51*, 3878–3894.

Chung, C.W., Dean, A.W., Woolven, J.M., and Bamborough, P. (2012). Fragment-based discovery of bromodomain inhibitors part 1: inhibitor binding modes and implications for lead discovery. J. Med. Chem. *55*, 576–586.

Clackson, T., and Wells, J.A. (1995). A hot spot of binding energy in a hormone-receptor interface. Science *267*, 383–386.

Coyne, A.G., Scott, D.E., and Abell, C. (2010). Drugging challenging targets using fragment-based approaches. Curr. Opin. Chem. Biol. *14*, 299–307.

Cummings, C.G., and Hamilton, A.D. (2010). Disrupting protein–protein interactions with non-peptidic, small molecule α-helix mimetics. Curr. Opin. Chem. Biol. *14*, 341–346.

Damm, K.L., and Carlson, H.A. (2007). Exploring experimental sources of multiple protein conformations in structure-based drug design. J. Am. Chem. Soc. *129*, 8225–8235.

Davis, M.I., Hunt, J.P., Herrgard, S., Ciceri, P., Wodicka, L.M., Pallares, G., Hocker, M., Treiber, D.K., and Zarrinkar, P.P. (2011). Comprehensive analysis of kinase inhibitor selectivity. Nat. Biotechnol. *29*, 1046–1051.

Demerdash, O.N., Daily, M.D., and Mitchell, J.C. (2009). Structure-based predictive models for allosteric hot spots. PLoS Comput. Biol. *5*, e1000531.

Dias, D.M., and Ciulli, A. (2014). NMR approaches in structure-based lead discovery: recent developments and new frontiers for targeting multi-protein complexes. Prog. Biophys. Mol. Biol. *116*, 101–112.

Dömling, A. (2008). Small molecular weight protein-protein interaction antagonists—an insurmountable challenge? Curr. Opin. Chem. Biol. *12*, 281–291.

Douse, C.H., Vrielink, N., Wenlin, Z., Cota, E., and Tate, E.W. (2015). Targeting a dynamic protein-protein interaction: fragment screening against the malaria myosin A motor complex. ChemMedChem *10*, 134–143.

Edfeldt, F.N., Folmer, R.H., and Breeze, A.L. (2011). Fragment screening to predict druggability (ligandability) and lead discovery success. Drug Discov. Today *16*, 284–287.

Ewing, T.J., and Kuntz, I.D. (1997). Critical evaluation of search algorithms for automated molecular docking and database screening. J. Comput. Chem. *18*, 1175–1189.

Fader, L.D., Malenfant, E., Parisien, M., Carson, R.J., Bilodeau, F., Landry, S., Pesant, M., Brochu, C., Morin, S., and Chabot, C. (2014). Discovery of BI 224436, a Non-Catalytic Site Integrase Inhibitor (NCINI) of HIV-1. ACS Med. Chem. Lett. *5*, 422–427.

Fan, H., Irwin, J.J., Webb, B.M., Klebe, G., Shoichet, B.K., and Sali, A. (2009). Molecular docking screens using comparative models of proteins. J. Chem. Inf. Model. *49*, 2512–2527.

Fernández-Recio, J. (2011). Prediction of protein binding sites and hot spots. Wiley Interdiscip. Rev. Comput. Mol. Sci. *1*, 680–698.

Fernandez-Recio, J., Totrov, M., and Abagyan, R. (2004). Identification of protein-protein interaction sites from docking energy landscapes. J. Mol. Biol. *335*, 843–865.

Filippakopoulos, P., Qi, J., Picaud, S., Shen, Y., Smith, W.B., Fedorov, O., Morse, E.M., Keates, T., Hickman, T.T., and Felletar, I. (2010). Selective inhibition of BET bromodomains. Nature *468*, 1067–1073.

Fischer, T., Arunachalam, K., Bailey, D., Mangual, V., Bakhru, S., Russo, R., Huang, D., Paczkowski, M., Lalchandani, V., and Ramachandra, C. (2003). The binding interface database (BID): a compilation of amino acid hot spots in protein interfaces. Bioinformatics *19*, 1453–1454.

Fjell, C.D., Jenssen, H., Hilpert, K., Cheung, W.A., Pante, N., Hancock, R.E., and Cherkasov, A. (2009). Identification of novel antibacterial peptides by chemoinformatics and machine learning. J. Med. Chem. *52*, 2006–2015.

Foster, T.J., MacKerell, A.D., and Guvench, O. (2012). Balancing target flexibility and target denaturation in computational fragment-based inhibitor discovery. J. Comput. Chem. *33*, 1880–1891.

Freire, E. (2000). Can allosteric regulation be predicted from structure? Proc. Natl. Acad. Sci. USA *97*, 11680–11682.

Fry, D.C. (2006). Protein-protein interactions as targets for small molecule drug discovery. Biopolymers *84*, 535–552.

Fry, D., Huang, K.S., Di Lello, P., Mohr, P., Müller, K., So, S.S., Harada, T., Stahl, M., Vu, B., and Mauser, H. (2013). Design of libraries targeting protein-protein interfaces. ChemMedChem *8*, 726–732.

Fuller, J.C., Burgoyne, N.J., and Jackson, R.M. (2009). Predicting druggable binding sites at the protein-protein interface. Drug Discov. Today *14*, 155–161.

Galloway, W.R., Isidro-Llobet, A., and Spring, D.R. (2010). Diversity-oriented synthesis as a tool for the discovery of novel biologically active small molecules. Nat. Commun. *1*, 80.

Gao, J., Ma, R., Wang, W., Wang, N., Sasaki, R., Snyderman, D., Wu, J., and Ruan, K. (2014). Automated NMR fragment based screening identified a novel interface blocker to the LARG/RhoA complex. PLoS One *9*, e88098.

Geppert, T., Bauer, S., Hiss, J.A., Conrad, E., Reutlinger, M., Schneider, P., Weisel, M., Pfeiffer, B., Altmann, K.H., and Waibler, Z. (2012). Immunosuppressive small molecule discovered by structure-based virtual screening for inhibitors of protein-protein interactions. Angew. Chem. Int. Ed. Engl. *51*, 258–261.

Gleeson, M.P., Hersey, A., Montanari, D., and Overington, J. (2011). Probing the links between in vitro potency, ADMET and physicochemical parameters. Nat. Rev. Drug Discov. *10*, 197–208.

Gohlke, H., Kiel, C., and Case, D.A. (2003). Insights into protein-protein binding by binding free energy calculation and free energy decomposition for the Ras-Raf and Ras-RalGDS complexes. J. Mol. Biol. *330*, 891–913.

Grigoryev, Y. (2013). Stapled peptide to enter human testing, but affinity questions remain. Nat. Med. *19*, 120.

Guney, E., Tuncbag, N., Keskin, O., and Gursoy, A. (2008). HotSprint: database of computational hot spots in protein interfaces. Nucleic Acids Res. *36*, D662–D666.

Gynther, M., Laine, K., Ropponen, J., Leppanen, J., Mannila, A., Nevalainen, T., Savolainen, J., Jarvinen, T.T., and Rautio, J. (2008). Large neutral amino acid transporter enables brain drug delivery via prodrugs. J. Med. Chem. *51*, 932–936.

Gynther, M., Ropponen, J., Laine, K., Leppanen, J., Haapakoski, P., Peura, L., Jarvinen, T., and Rautio, J. (2009). Glucose promoiety enables glucose transporter mediated brain uptake of ketoprofen and indomethacin prodrugs in rats. J. Med. Chem. *52*, 3348–3353.

Haider, M.K., and Huggins, D.J. (2013). Combining solvent thermodynamic profiles with functionality maps of the Hsp90 binding site to predict the displacement of water molecules. J. Chem. Inf. Model. *53*, 2571–2586.

Hajduk, P.J. (2006). Fragment-based drug design: how big is too big? J. Med. Chem. *49*, 6972–6976.

Hajduk, P.J., Huth, J.R., and Tse, C. (2005). Predicting protein druggability. Drug Discov. Today *10*, 1675–1682.

Halgren, T.A. (2009). Identifying and characterizing binding sites and assessing druggability. J. Chem. Inf. Model. *49*, 377–389.

Hamon, V., Brunel, J.M., Combes, S., Basse, M.J., Roche, P., and Morelli, X. (2013). 2P2Ichem: focused chemical libraries dedicated to orthosteric modulation of protein-protein interactions. Med. Chem. Commun. *4*, 797–809.

 ©2015 Elsevier Ltd All rights reserved

Harris, J.C., Hill, R.D., Sheppard, D.W., Slater, M.J., and Stouten, P.F. (2011). The design and application of target-focused compound libraries. Comb. Chem. High Throughput Screen. *14*, 521–531.

Higueruelo, A.P., Schreyer, A., Bickerton, G.R.J., Pitt, W.R., Groom, C.R., and Blundell, T.L. (2009). Atomic interactions and profile of small molecules disrupting protein-protein interfaces: the TIMBAL database. Chem. Biol. Drug Des. *74*, 457–467.

Higueruelo, A.P., Jubb, H., and Blundell, T.L. (2013). Protein-protein interactions as druggable targets: recent technological advances. Curr. Opin. Pharmacol. *13*, 791–796.

Holvey, R.S., Valkov, E., Neal, D., Stewart, M., and Abell, C. (2015). Selective targeting of the TPX2 site of importin-α using fragment-based ligand design. ChemMedChem. Published online April 20, 2015. http://dx.doi.org/10.1002/cmdc.201500014.

Hood, L., and Perlmutter, R.M. (2004). The impact of systems approaches on biological problems in drug discovery. Nat. Biotechnol. *22*, 1215–1217.

Hopkins, A.L. (2008). Network pharmacology: the next paradigm in drug discovery. Nat. Chem. Biol. *4*, 682–690.

Hopkins, A.L., Groom, C.R., and Alex, A. (2004). Ligand efficiency: a useful metric for lead selection. Drug Discov. Today *9*, 430–431.

Hopkins, A.L., Keserü, G.M., Leeson, P.D., Rees, D.C., and Reynolds, C.H. (2014). The role of ligand efficiency metrics in drug discovery. Nat. Rev. Drug Discov. *13*, 105–121.

Huggins, D.J., and Payne, M.C. (2013). Assessing the accuracy of inhomogeneous fluid solvation theory in predicting hydration free energies of simple solutes. J. Phys. Chem. B *117*, 8232–8244.

Huggins, D.J., McKenzie, G., Robinson, D., Narváez, A., Hardwick, B., Roberts-Thomson, M., Venkitaraman, A., Grant, G., and Payne, M. (2010). Computational analysis of phosphopeptide binding to the polo-box domain of the mitotic kinase PLK1 using molecular dynamics simulation. PLoS Comput. Biol. *6*, http://dx.doi.org/10.1371/journal.pcbi.1000880.

Huggins, D.J., Venkitaraman, A.R., and Spring, D.R. (2011). Rational methods for the selection of diverse screening compounds. ACS Chem. Biol. *6*, 208–217.

Hung, A.W., Ramek, A., Wang, Y., Kaya, T., Wilson, J.A., Clemons, P.A., and Young, D.W. (2011). Route to three-dimensional fragments using diversity-oriented synthesis. Proc. Natl. Acad. Sci. USA *108*, 6799–6804.

Janin, J. (2010). Protein-protein docking tested in blind predictions: the CAPRI experiment. Mol. Biosyst. *6*, 2351–2362.

Johnson, D.K., and Karanicolas, J. (2013). Druggable protein interaction sites are more predisposed to surface pocket formation than the rest of the protein surface. PLoS Comput. Biol. *9*, e1002951.

Johnson, T.W., Dress, K.R., and Edwards, M. (2009). Using the Golden Triangle to optimize clearance and oral absorption. Bioorg. Med. Chem. Lett. *19*, 5560–5564.

Jones, G., Willett, P., and Glen, R.C. (1995). Molecular recognition of receptor sites using a genetic algorithm with a description of desolvation. J. Mol. Biol. *245*, 43–53.

Jose, R.A., Voet, A., Broos, K., Jakobi, A.J., Bruylants, G., Egle, B., Zhang, K.Y., De Maeyer, M., Deckmyn, H., and De Borggraeve, W.M. (2012). An integrated fragment based screening approach for the discovery of small molecule modulators of the VWF-GPIbalpha interaction. Chem. Commun. (Camb.) *48*, 11349–11351.

Kim, D.E., Chivian, D., and Baker, D. (2004). Protein structure prediction and analysis using the Robetta server. Nucleic Acids Res. *32*, W526–W531.

Koes, D., Khoury, K., Huang, Y., Wang, W., Bista, M., Popowicz, G.M., Wolf, S., Holak, T.A., Dömling, A., and Camacho, C.J. (2012). Enabling large-scale design, synthesis and validation of small molecule protein-protein antagonists. PLoS one *7*, e32839.

Korb, O., Olsson, T.S., Bowden, S.J., Hall, R.J., Verdonk, M.L., Liebeschuetz, J.W., and Cole, J.C. (2012). Potential and limitations of ensemble docking. J. Chem. Inf. Model. *52*, 1262–1274.

Kreeger, P.K., and Lauffenburger, D.A. (2010). Cancer systems biology: a network modeling perspective. Carcinogenesis *31*, 2–8.

Krüger, D.M., and Gohlke, H. (2010). DrugScorePPI webserver: fast and accurate in silico alanine scanning for scoring protein–protein interactions. Nucleic Acids Res. *38*, W480–W486.

Kuenemann, M.A., Bourbon, L.M., Labbe, C.M., Villoutreix, B.O., and Sperandio, O. (2014). Which three-dimensional characteristics make efficient inhibitors of protein-protein interactions? J. Chem. Inf. Model. *54*, 3067–3079.

Labbé, C.M., Laconde, G., Kuenemann, M.A., Villoutreix, B.O., and Sperandio, O. (2013). iPPI-DB: A manually curated and interactive database of small non-peptide inhibitors of protein-protein interactions. Drug Discov. Today *18*, 958–968.

Landon, M.R., Lancia, D.R., Yu, J., Thiel, S.C., and Vajda, S. (2007). Identification of hot spots within druggable binding regions by computational solvent mapping of proteins. J. Med. Chem. *50*, 1231–1240.

Lao, B.B., Grishagin, I., Mesallati, H., Brewer, T.F., Olenyuk, B.Z., and Arora, P.S. (2014). In vivo modulation of hypoxia-inducible signaling by topographical helix mimetics. Proc. Natl. Acad. Sci. USA *111*, 7531–7536.

Laraia, L., and Spring, D.R. (2013). Chemical library screening approaches to aid the design of protein–protein inhibitors. In Understanding and Exploiting Protein-Protein Interactions as Drug Targets, G. Zinzalla, ed. (Future Science), pp. 32–45.

Lau, Y.H., de Andrade, P., Quah, S.-T., Rossmann, M., Laraia, L., Sköld, N., Sum, T.J., Rowling, P.J., Joseph, T.L., Verma, C., et al. (2014). Functionalised staple linkages for modulating the cellular activity of stapled peptides. Chem. Sci. *5*, 1804–1809.

Leader, B., Baca, Q.J., and Golan, D.E. (2008). Protein therapeutics: a summary and pharmacological classification. Nat. Rev. Drug Discov. *7*, 21–39.

Lee, J.H., Zhang, Q., Jo, S., Chai, S.C., Oh, M., Im, W., Lu, H., and Lim, H.S. (2011). Novel pyrrolopyrimidine-based alpha-helix mimetics: cell-permeable inhibitors of protein-protein interactions. J. Am. Chem. Soc. *133*, 676–679.

Leeson, P.D., and Springthorpe, B. (2007). The influence of drug-like concepts on decision-making in medicinal chemistry. Nat. Rev. Drug Discov. *6*, 881–890.

Lexa, K.W., and Carlson, H.A. (2010). Full protein flexibility is essential for proper hot-spot mapping. J. Am. Chem. Soc. *133*, 200–202.

Lexa, K.W., and Carlson, H.A. (2013). Improving protocols for protein mapping through proper comparison to crystallography data. J. Chem. Inf. Model. *53*, 391–402.

Li, Z., and Lazaridis, T. (2006). Thermodynamics of buried water clusters at a protein-ligand binding interface. J. Phys. Chem. B *110*, 1464–1475.

Liskamp, R.M., Rijkers, D.T., and Bakker, S.E. (2008). Bioactive macrocyclic peptides and peptide mimics. In Modern Supramolecular Chemistry: Strategies for Macrocycle Synthesis, F. Diederich, P.J. Stang, and R.R. Tykwinski, eds. (Wiley-VCH), pp. 1–27.

Liu, T., Lin, Y., Wen, X., Jorissen, R.N., and Gilson, M.K. (2007). BindingDB: a web-accessible database of experimentally determined protein-ligand binding affinities. Nucleic Acids Res. *35*, D198–D201.

Lund, G., Dudkin, S., Borkin, D., Ni, W., Grembecka, J., and Cierpicki, T. (2015). Inhibition of CDC25B phosphatase through disruption of protein-protein interaction. ACS Chem. Biol. *10*, 390–394.

Marcaurelle, L.A., Johannes, C., Yohannes, D., Tillotson, B.P., and Mann, D. (2009). Diversity-oriented synthesis of a cytisine-inspired pyridone library leading to the discovery of novel inhibitors of Bcl-2. Bioorg. Med. Chem. Lett. *19*, 2500–2503.

Martins, S.A., Perez, M.A., Moreira, I.S., Sousa, S.r.F., Ramos, M., and Fernandes, P. (2013). Computational alanine scanning mutagenesis: MM-PBSA vs TI. J. Chem. Theor. Comput. *9*, 1311–1319.

Matsuno, K., Masuda, Y., Uehara, Y., Sato, H., Muroya, A., Takahashi, O., Yokotagawa, T., Furuya, T., Okawara, T., and Otsuka, M. (2010). Identification of a new series of STAT3 inhibitors by virtual screening. ACS Med. Chem. Lett. *1*, 371–375.

Maurer, T., Garrenton, L.S., Oh, A., Pitts, K., Anderson, D.J., Skelton, N.J., Fauber, B.P., Pan, B., Malek, S., Stokoe, D., et al. (2012). Small-molecule ligands bind to a distinct pocket in Ras and inhibit SOS-mediated nucleotide exchange activity. Proc. Natl. Acad. Sci. USA *109*, 5299–5304.

Metz, A., Pfleger, C., Kopitz, H., Pfeiffer-Marek, S., Baringhaus, K.H., and Gohlke, H. (2012). Hot spots and transient pockets: predicting the determinants of small-molecule binding to a protein-protein interface. J. Chem. Inf. Model. *52*, 120–133.

Miyashita, N., Straub, J.E., Thirumalai, D., and Sugita, Y. (2009). Transmembrane structures of amyloid precursor protein dimer predicted by replica-exchange molecular dynamics simulations. J. Am. Chem. Soc. *131*, 3438–3439.

Moellering, R.E., Cornejo, M., Davis, T.N., Del Bianco, C., Aster, J.C., Blacklow, S.C., Kung, A.L., Gilliland, D.G., Verdine, G.L., and Bradner, J.E. (2009). Direct inhibition of the NOTCH transcription factor complex. Nature *462*, 182–188.

Molzan, M., Weyand, M., Rose, R., and Ottmann, C. (2012). Structural insights of the MLF1/14-3-3 interaction. FEBS J. *279*, 563–571.

Moore, C.D., Wu, H., Bolanos, B., Bergqvist, S., Brooun, A., Pauly, T., and Nowlin, D. (2009). Structural and biophysical characterization of XIAP BIR3 G306E mutant: insights in protein dynamics and application for fragment-based drug design. Chem. Biol. Drug Des. *74*, 212–223.

Moreira, I.S., Fernandes, P.A., and Ramos, M.J. (2007). Computational alanine scanning mutagenesis—an improved methodological approach. J. Comput. Chem. *28*, 644–654.

Morelli, X., Bourgeas, R., and Roche, P. (2011). Chemical and structural lessons from recent successes in protein-protein interaction inhibition (2P2I). Curr. Opin. Chem. Biol. *15*, 475–481.

Morley, A.D., Pugliese, A., Birchall, K., Bower, J., Brennan, P., Brown, N., Chapman, T., Drysdale, M., Gilbert, I.H., Hoelder, S., et al. (2013). Fragment-based hit identification: thinking in 3D. Drug Discov. Today *18*, 1221–1227.

Mullard, A. (2012). Protein-protein interaction inhibitors get into the groove. Nat. Rev. Drug Discov. *11*, 173–175.

Murphy, N.S., Prabhakaran, P., Azzarito, V., Plante, J.P., Hardie, M.J., Kilner, C.A., Warriner, S.L., and Wilson, A.J. (2013). Solid-phase methodology for synthesis of O-alkylated aromatic oligoamide inhibitors of α-helix-mediated protein-protein interactions. Chem-Eur J. *19*, 5546–5550.

Negi, S.S., and Braun, W. (2007). Statistical analysis of physical-chemical properties and prediction of protein-protein interfaces. J. Mol. Model. *13*, 1157–1167.

Neugebauer, A., Hartmann, R.W., and Klein, C.D. (2007). Prediction of protein-protein interaction inhibitors by chemoinformatics and machine learning methods. J. Med. Chem. *50*, 4665–4668.

Noble, R.L., Gout, P.W., Wijcik, L.L., Hebden, H.F., and Beer, C.T. (1977). The distribution of [^{3}H] vinblastine in tumor and host tissues of Nb rats bearing a transplantable lymphoma which is highly sensitive to the alkaloid. Cancer Res. *37*, 1455–1460.

Oltersdorf, T., Elmore, S.W., Shoemaker, A.R., Armstrong, R.C., Augeri, D.J., Belli, B.A., Bruncko, M., Deckwerth, T.L., Dinges, J., Hajduk, P.J., et al. (2005). An inhibitor of Bcl-2 family proteins induces regression of solid tumours. Nature *435*, 677–681.

Overington, J.P., Al-Lazikani, B., and Hopkins, A.L. (2006). How many drug targets are there? Nat. Rev. Drug Discov. *5*, 993–996.

Pagliaro, L., Felding, J., Audouze, K., Nielsen, S.J., Terry, R.B., Krog-Jensen, C., and Butcher, S. (2004). Emerging classes of protein-protein interaction inhibitors and new tools for their development. Curr. Opin. Chem. Biol. *8*, 442–449.

Parai, M.K., Huggins, D.J., Cao, H., Nalam, M.N.L., Ali, A., Schiffer, C.A., Tidor, B., and Rana, T.M. (2012). Design, synthesis, and biological and structural evaluations of novel HIV-1 protease inhibitors to combat drug resistance. J. Med. Chem. *55*, 6328–6341.

Patrone, J.D., Kennedy, J.P., Frank, A.O., Feldkamp, M.D., Vangamudi, B., Pelz, N.F., Rossanese, O.W., Waterson, A.G., Chazin, W.J., and Fesik, S.W. (2013). Discovery of protein-protein interaction inhibitors of replication protein A. ACS Med. Chem. Lett. *4*, 601–605.

Pfaff, S.J., Chimenti, M.S., Kelly, M.J., and Arkin, M.R. (2015). Biophysical methods for identifying fragment-based inhibitors of protein-protein interactions. Methods Mol. Biol. *1278*, 587–613.

Pitre, S., Dehne, F., Chan, A., Cheetham, J., Duong, A., Emili, A., Gebbia, M., Greenblatt, J., Jessulat, M., and Krogan, N. (2006). PIPE: a protein-protein interaction prediction engine based on the re-occurring short polypeptide sequences between known interacting protein pairs. BMC Bioinformatics *7*, 365.

Pommier, Y., and Marchand, C. (2012). Interfacial inhibitors: targeting macromolecular complexes. Nat. Rev. Drug Discov. *11*, 25–36.

Raman, E.P., Yu, W., Guvench, O., and MacKerell, A.D., Jr. (2011). Reproducing crystal binding modes of ligand functional groups using Site-Identification by Ligand Competitive Saturation (SILCS) simulations. J. Chem. Inf. Model. *51*, 877–896.

Reynès, C., Host, H., Camproux, A.-C., Laconde, G., Leroux, F., Mazars, A., Deprez, B., Fahraeus, R., Villoutreix, B.O., and Sperandio, O. (2010). Designing focused chemical libraries enriched in protein-protein interaction inhibitors using machine-learning methods. PLoS Comput. Biol. *6*, e1000695.

Rhodes, N., Heerding, D.A., Duckett, D.R., Eberwein, D.J., Knick, V.B., Lansing, T.J., McConnell, R.T., Gilmer, T.M., Zhang, S.-Y., and Robell, K. (2008). Characterization of an Akt kinase inhibitor with potent pharmacodynamic and antitumor activity. Cancer Res. *68*, 2366–2374.

Riniker, S., Christ, C.D., Hansen, H.S., Hünenberger, P.H., Oostenbrink, C., Steiner, D., and van Gunsteren, W.F. (2011). Calculation of relative free energies for ligand-protein binding, solvation, and conformational transitions using the GROMOS software. J. Phys. Chem. B *115*, 13570–13577.

Ripphausen, P., Nisius, B., Peltason, L., and Bajorath, J. (2010). Quo vadis, virtual screening? A comprehensive survey of prospective applications. J. Med. Chem. *53*, 8461–8467.

Roepe, P.D. (2001). A peptide needle in a signaling haystack. Nat. Genet. *27*, 6–7.

Rouhana, J., Hoh, F., Estaran, S., Henriquet, C., Boublik, Y., Kerkour, A., Trouillard, R., Martinez, J., Pugniere, M., Padilla, A., et al. (2013). Fragment-based identification of a locus in the Sec7 domain of Arno for the design of protein-protein interaction inhibitors. J. Med. Chem. *56*, 8497–8511.

Ruffner, H., Bauer, A., and Bouwmeester, T. (2007). Human protein-protein interaction networks and the value for drug discovery. Drug Discov. Today *12*, 709–716.

Sathish, J.G., Sethu, S., Bielsky, M.-C., de Haan, L., French, N.S., Govindappa, K., Green, J., Griffiths, C.E., Holgate, S., and Jones, D. (2013). Challenges and approaches for the development of safer immunomodulatory biologics. Nat. Rev. Drug Discov. *12*, 306–324.

Schames, J.R., Henchman, R.H., Siegel, J.S., Sotriffer, C.A., Ni, H., and McCammon, J.A. (2004). Discovery of a novel binding trench in HIV integrase. J. Med. Chem. *47*, 1879–1881.

Schneider, G. (2012). Designing the molecular future. J. Comput. Aided Mol. Des. *26*, 115–120.

Scior, T., Bender, A., Tresadern, G., Medina-Franco, J.L., Martínez-Mayorga, K., Langer, T., Cuanalo-Contreras, K., and Agrafiotis, D.K. (2012). Recognizing pitfalls in virtual screening: a critical review. J. Chem. Inf. Model. *52*, 867–881.

Scott, M.S., and Barton, G.J. (2007). Probabilistic prediction and ranking of human protein-protein interactions. BMC Bioinformatics *8*, 239.

Scott, D.E., Ehebauer, M.T., Pukala, T., Marsh, M., Blundell, T.L., Venkitaraman, A.R., Abell, C., and Hyvönen, M. (2013). Using a fragment-based approach to target protein-protein interactions. ChemBioChem *14*, 332–342.

Shaginian, A., Whitby, L.R., Hong, S., Hwang, I., Farooqi, B., Searcey, M., Chen, J., Vogt, P.K., and Boger, D.L. (2009). Design, synthesis, and evaluation of an alpha-helix mimetic library targeting protein-protein interactions. J. Am. Chem. Soc. *131*, 5564–5572.

Sheridan, R.P., Maiorov, V.N., Holloway, M.K., Cornell, W.D., and Gao, Y.-D. (2010). Drug-like density: a method of quantifying the "bindability" of a protein target based on a very large set of pockets and drug-like ligands from the Protein Data Bank. J. Chem. Inf. Model. *50*, 2029–2040.

Tan, Y.S., Śledź, P., Lang, S., Stubbs, C.J., Spring, D.R., Abell, C., and Best, R.B. (2012). Using ligand-mapping simulations to design a ligand selectively

 ©2015 Elsevier Ltd All rights reserved

targeting a cryptic surface pocket of polo-like kinase 1. Angew. Chem. Int. Ed. Engl. *124*, 10225–10228.

Teague, S.J., Davis, A.M., Leeson, P.D., and Oprea, T. (1999). The design of leadlike combinatorial libraries. Angew. Chem. Int. Ed. Engl. *38*, 3743–3748.

Thorn, K.S., and Bogan, A.A. (2001). ASEdb: a database of alanine mutations and their effects on the free energy of binding in protein interactions. Bioinformatics *17*, 284–285.

Totrov, M., and Abagyan, R. (2008). Flexible ligand docking to multiple receptor conformations: a practical alternative. Curr. Opin. Struct. Biol. *18*, 178–184.

Tse, C., Shoemaker, A.R., Adickes, J., Anderson, M.G., Chen, J., Jin, S., Johnson, E.F., Marsh, K.C., Mitten, M.J., and Nimmer, P. (2008). ABT-263: a potent and orally bioavailable Bcl-2 family inhibitor. Cancer Res. *68*, 3421–3428.

Tuncbag, N., Gursoy, A., and Keskin, O. (2009). Identification of computational hot spots in protein interfaces: combining solvent accessibility and inter-residue potentials improves the accuracy. Bioinformatics *25*, 1513–1520.

Tuncbag, N., Keskin, O., and Gursoy, A. (2010). HotPoint: hot spot prediction server for protein interfaces. Nucleic Acids Res. *38*, W402–W406.

Tuncbag, N., Keskin, O., Nussinov, R., and Gursoy, A. (2012). Fast and accurate modeling of protein-protein interactions by combining template-interface-based docking with flexible refinement. Proteins *80*, 1239–1249.

Valkov, E., Sharpe, T., Marsh, M., Greive, S., and Hyvonen, M. (2012). Targeting protein-protein interactions and fragment-based drug discovery. Top. Curr. Chem. *317*, 145–179.

Van Molle, I., Thomann, A., Buckley, D.L., So, E.C., Lang, S., Crews, C.M., and Ciulli, A. (2012). Dissecting fragment-based lead discovery at the von Hippel-Lindau protein:hypoxia inducible factor 1alpha protein-protein interface. Chem. Biol. *19*, 1300–1312.

Villoutreix, B.O., Kuenemann, M.A., Poyet, J.L., Bruzzoni-Giovanelli, H., Labbe, C., Lagorce, D., Sperandio, O., and Miteva, M.A. (2014). Drug-like protein-protein interaction modulators: challenges and opportunities for drug discovery and chemical biology. Mol. Inform. *33*, 414–437.

Vu, B., Wovkulich, P., Pizzolato, G., Lovey, A., Ding, Q., Jiang, N., Liu, J.-J., Zhao, C., Glenn, K., and Wen, Y. (2013). Discovery of RG7112: a small-molecule MDM2 inhibitor in clinical development. ACS Med. Chem. Lett. *4*, 466–469.

Walensky, L.D., and Bird, G.H. (2014). Hydrocarbon-stapled peptides: principles, practice, and progress. J. Med. Chem. *57*, 6275–6288.

Wang, L., Deng, Y., Knight, J.L., Wu, Y., Kim, B., Sherman, W., Shelley, J.C., Lin, T., and Abel, R. (2013). Modeling local structural rearrangements using FEP/REST: application to relative binding affinity predictions of CDK2 inhibitors. J. Chem. Theor. Comput. *9*, 1282–1293.

Wells, J.A., and McClendon, C.L. (2007). Reaching for high-hanging fruit in drug discovery at protein-protein interfaces. Nature *450*, 1001–1009.

Whitby, L.R., and Boger, D.L. (2012). Comprehensive peptidomimetic libraries targeting protein-protein interactions. Acc. Chem. Res. *45*, 1698–1709.

Wilson, C.G., and Arkin, M.R. (2013). Probing structural adaptivity at PPI interfaces with small molecules. Drug Discov. Today *10*, e501–e508.

Winter, A., Higueruelo, A.P., Marsh, M., Sigurdardottir, A., Pitt, W.R., and Blundell, T.L. (2012). Biophysical and computational fragment-based approaches to targeting protein-protein interactions: applications in structure-guided drug discovery. Q. Rev. Biophys. *45*, 383–426.

Xu, X., Leo, C., Jang, Y., Chan, E., Padilla, D., Huang, B.C., Lin, T., Gururaja, T., Hitoshi, Y., and Lorens, J.B. (2001). Dominant effector genetics in mammalian cells. Nat. Genet. *27*, 23–29.

Yildirim, M.A., Goh, K.I., Cusick, M.E., Barabasi, A.L., and Vidal, M. (2007). Drug-target network. Nat. Biotechnol. *25*, 1119–1126.

Yin, Z., Whittell, L.R., Wang, Y., Jergic, S., Liu, M., Harry, E.J., Dixon, N.E., Beck, J.L., Kelso, M.J., and Oakley, A.J. (2014). Discovery of lead compounds targeting the bacterial sliding clamp using a fragment-based approach. J. Med. Chem. *57*, 2799–2806.

Zerbe, B.S., Hall, D.R., Vajda, S., Whitty, A., and Kozakov, D. (2012). Relationship between hot spot residues and ligand binding hot spots in protein-protein interfaces. J. Chem. Inf. Model. *52*, 2236–2244.

Zhang, J., Adrián, F.J., Jahnke, W., Cowan-Jacob, S.W., Li, A.G., Iacob, R.E., Sim, T., Powers, J., Dierks, C., and Sun, F. (2010). Targeting Bcr-Abl by combining allosteric with ATP-binding-site inhibitors. Nature *463*, 501–506.

Zhong, M., Gadek, T.R., Bui, M., Shen, W., Burnier, J., Barr, K.J., Hanan, E.J., Oslob, J.D., Yu, C.H., and Zhu, J. (2012). Discovery and development of potent LFA-1/ICAM-1 antagonist SAR 1118 as an ophthalmic solution for treating dry eye. ACS Med. Chem. Lett. *3*, 203–206.

Zinzalla, G., and Thurston, D.E. (2009). Targeting protein-protein interactions for therapeutic intervention: a challenge for the future. Future Med. Chem. *1*, 65–93.

Chemistry & Biology, Volume *22*

Supplemental Information

Overcoming Chemical, Biological, and Computational Challenges in the Development of Inhibitors Targeting Protein-Protein Interactions

Luca Laraia, Grahame McKenzie, David R. Spring, Ashok R. Venkitaraman, and David J. Huggins

<u>**Supporting Information**</u>

Supplemental Data

Figure S1, Related to Figure 1

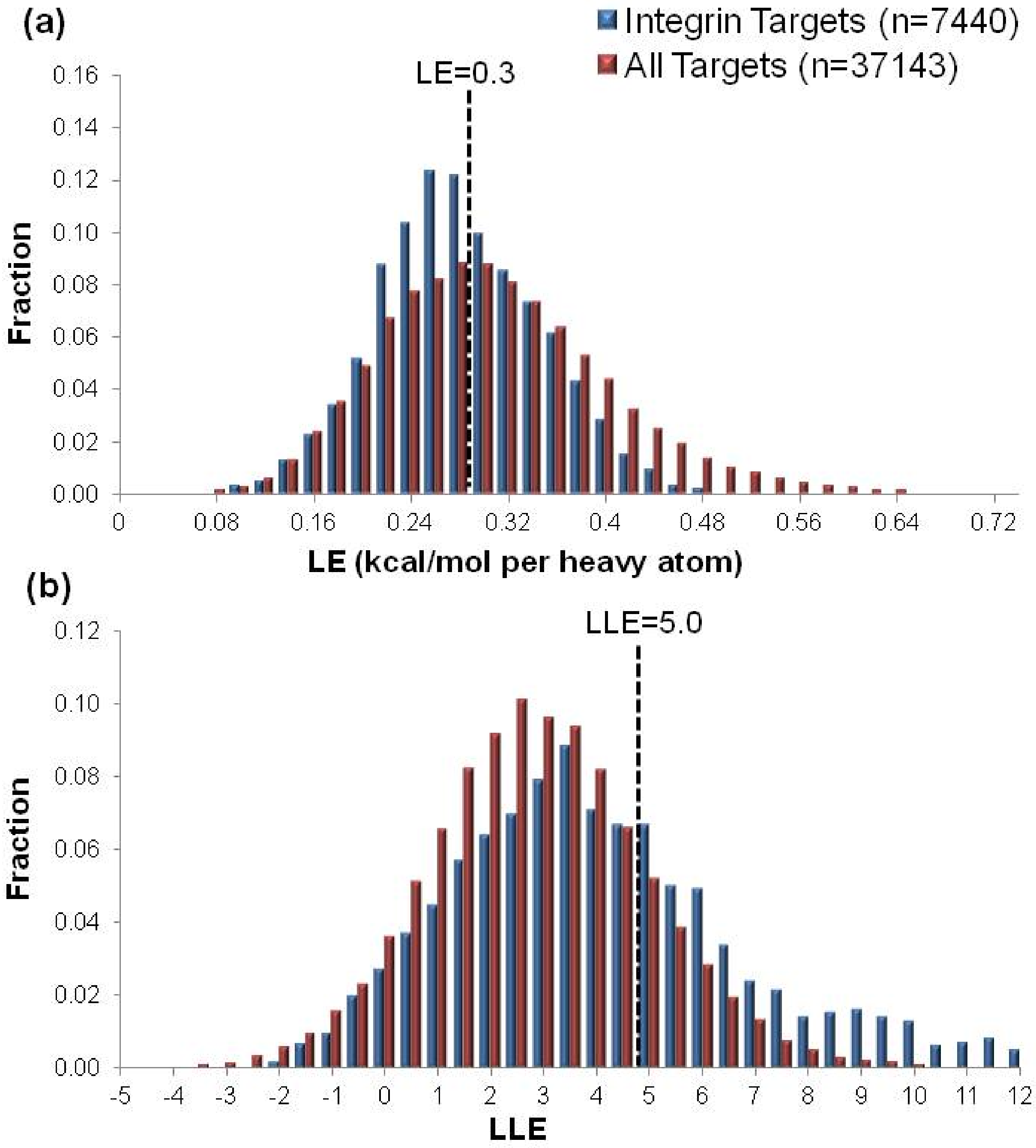

Figure S1 - Bar graphs showing the distributions of (a) LE and (b) LLE using IC_{50} data for 7440 integrin inhibitors in the TIMBAL database and 37143 small molecule inhibitors in the curated portion of the BindingDB database. Heavy atom counts and cLogP values were computed using Schrödinger's Qikprop and the small molecules were prepared using Schrödinger's Ligprep. The average LE for the integrin inhibitors is 0.29 kcal/mol per heavy atom, compared with an average of 0.23 kcal/mol per heavy atom for the other PPI inhibitors in TIMBAL and 0.32 kcal/mol per heavy atom for inhibitors in the BindingDB. The average LLE for the PPI inhibitors studied was 4.38, compared with an average of 1.32 for the other PPI inhibitors in TIMBAL and 3.12 for inhibitors in the BindingDB. The percentages of integrin inhibitors in the TIMBAL database, other PPI inhibitors in the TIMBAL database and inhibitors in the BindingDB passing the LE filter are 42.9%, 14.5%, and 54.8% respectively. The percentages of integrin inhibitors in the TIMBAL database, other PPI inhibitors in the TIMBAL database and inhibitors in the BindingDB passing the LLE filter are 35.8%, 4.5%, and 17.4% respectively.

Table S1

Technique	Acronym	Description	Software
Molecular Mechanics - Generalized Born Surface Area (Massova and Kollman, 2000)	MM-GBSA	A method for computing the free energy difference between two states by computing the free energies using forcefield based energies and a Generalized Born implicit solvent model.	NAMD (Phillips, et al., 2005)<br>AMBER (Case, et al., 2005)<br>GROMACS (Van Der Spoel, et al., 2005)<br>CHARMM (Brooks, et al., 2009)<br>GROMOS (Christen, et al., 2005)<br>Desmond (Bowers, et al., 2006)
Free Energy Perturbation (Liu, et al., 2012)	FEP	A statistical mechanical method for computing the free energy difference between two states by calculating the sum of free energy changes for a series of small steps along the pathway between them.	NAMD,<br>AMBER,<br>GROMACS,<br>CHARMM,<br>GROMOS,<br>Desmond
Thermodynamic Integration (Lawrenz, et al., 2012)	TI	A statistical mechanical method for computing the free energy difference between two states by integrating over the enthalpy changes along the pathway between them.	NAMD,<br>AMBER,<br>GROMACS,<br>CHARMM,<br>GROMOS,<br>Desmond
Inhomogeneous Fluid Solvation Theory (Lazaridis, 2000)	IFST	A statistical mechanical method for computing the free energy difference between two states by calculating the effect of the change on the surrounding solvent.	STOW (Li and Lazaridis, 2012)<br>WaterMap (Young, et al., 2007)<br>GIST (Nguyen, et al., 2012)

Virtual Screening (Scior, et al., 2012)	VS	A method for identifying potential inhibitors of a given protein from computational analysis of a large library of molecules. The most common approaches are shape-based screening, pharmacophore screening and molecular docking,	Glide (Hippertt, et al., 2001)<br>GOLD (Verdonk, et al., 2003)<br>DOCK (Moustakas, et al., 2006)<br>ROCS (Rush, et al., 2005)
Replica Exchange Molecular Dynamics (Rao and Caflisch, 2003)	REMD	A technique that enhances MD sampling by performing parallel simulations of a system at multiple temperatures and allowing the different systems to exchange.	NAMD,<br>AMBER,<br>GROMACS,<br>CHARMM,<br>GROMOS,<br>Desmond

Table S1 - A description of some of the computational techniques discussed in this paper.

Supplementary References

Bowers, K.J., Chow, E., Xu, H., Dror, R.O., Eastwood, M.P., Gregersen, B.A., Klepeis, J.L., Kolossvary, I., Moraes, M.A., and Sacerdoti, F.D. (2006). Scalable algorithms for molecular dynamics simulations on commodity clusters. In *SC 2006 Conference, Proceedings of the ACM/IEEE*. (IEEE). pp. 43-43.

Brooks, B.R., Brooks, C.L., 3rd, Mackerell, A.D., Jr., Nilsson, L., Petrella, R.J., Roux, B., Won, Y., Archontis, G., Bartels, C., Boresch, S., et al. (2009). CHARMM: the biomolecular simulation program. J. Comput. Chem. 30, 1545-1614.

Case, D.A., Cheatham, T.E., Darden, T., Gohlke, H., Luo, R., Merz, K.M., Onufriev, A., Simmerling, C., Wang, B., and Woods, R.J. (2005). The Amber biomolecular simulation programs. J. Comput. Chem. 26, 1668-1688.

Christen, M., Hünenberger, P.H., Bakowies, D., Baron, R., Bürgi, R., Geerke, D.P., Heinz, T.N., Kastenholz, M.A., Kräutler, V., and Oostenbrink, C. (2005). The GROMOS software for biomolecular simulation: GROMOS05. J. Comput. Chem. 26, 1719-1751.

Hippertt, J., Rocha, A., Lana, C., Egydio-Silva, M., and Takeshita, T. (2001). Quartz plastic segregation and ribbon development in high-grade striped gneisses. J Struct Geol 23, 67-80.

Lawrenz, M., Wereszczynski, J., Ortiz-Sánchez, J.M., Nichols, S.E., and McCammon, J.A. (2012). Thermodynamic integration to predict host-guest binding affinities. J. Comput.-Aided Mol. Des. 26, 569-576.

Lazaridis, T. (2000). Solvent reorganization energy and entropy in hydrophobic hydration. J. Phys. Chem. B 104, 4964-4979.

Li, Z., and Lazaridis, T. (2012). Computing the thermodynamic contributions of interfacial water. Methods Mol. Biol. 819, 393-404.

Liu, P., Dehez, F., Cai, W.S., and Chipot, C. (2012). A Toolkit for the Analysis of Free-Energy Perturbation Calculations. J. Chem. Theory Comput. 8, 2606-2616.

Massova, I., and Kollman, P.A. (2000). Combined molecular mechanical and continuum solvent approach (MM-PBSA/GBSA) to predict ligand binding. Perspect. Drug Discovery Des. 18, 113-135.

Moustakas, D.T., Lang, P.T., Pegg, S., Pettersen, E., Kuntz, I.D., Brooijmans, N., and Rizzo, R.C. (2006). Development and validation of a modular, extensible docking program: DOCK 5. J. Comput.-Aided Mol. Des. 20, 601-619.

Nguyen, C.N., Young, T.K., and Gilson, M.K. (2012). Grid inhomogeneous solvation theory: Hydration structure and thermodynamics of the miniature receptor cucurbit[7]uril. J. Chem. Phys. 137, 044101.

Phillips, J.C., Braun, R., Wang, W., Gumbart, J., Tajkhorshid, E., Villa, E., Chipot, C., Skeel, R.D., Kale, L., and Schulten, K. (2005). Scalable molecular dynamics with NAMD. J. Comput. Chem. 26, 1781-1802.

Rao, F., and Caflisch, A. (2003). Replica exchange molecular dynamics simulations of reversible folding. The Journal of Chemical Physics 119, 4035.

Rush, T.S., Grant, J.A., Mosyak, L., and Nicholls, A. (2005). A shape-based 3-D scaffold hopping method and its application to a bacterial protein-protein interaction. J. Med. Chem. 48, 1489-1495.

Scior, T., Bender, A., Tresadern, G., Medina-Franco, J.L., Martínez-Mayorga, K., Langer, T., Cuanalo-Contreras, K., and Agrafiotis, D.K. (2012). Recognizing pitfalls in virtual screening: a critical review. J. Chem. Inf. Model. 52, 867-881.

Van Der Spoel, D., Lindahl, E., Hess, B., Groenhof, G., Mark, A.E., and Berendsen, H.J. (2005). GROMACS: fast, flexible, and free. J. Comput. Chem. 26, 1701-1718.
Verdonk, M.L., Cole, J.C., Hartshorn, M.J., Murray, C.W., and Taylor, R.D. (2003). Improved protein–ligand docking using GOLD. Proteins: Struct., Funct., Bioinf. 52, 609-623.
Young, T., Abel, R., Kim, B., Berne, B.J., and Friesner, R.A. (2007). Motifs for molecular recognition exploiting hydrophobic enclosure in protein-ligand binding. Proc. Natl. Acad. Sci. USA 104, 808-813.
